# Functional diversification of the potato R2R3 MYB anthocyanin activators AN1, MYBA1, and MYB113 and their interaction with basic helix-loop-helix cofactors

**DOI:** 10.1093/jxb/erw014

**Published:** 2016-02-16

**Authors:** Yuhui Liu, Kui Lin-Wang, Richard V. Espley, Li Wang, Hongyu Yang, Bin Yu, Andrew Dare, Erika Varkonyi-Gasic, Jing Wang, Junlian Zhang, Di Wang, Andrew C. Allan

**Affiliations:** ^1^Gansu Key Laboratory of Crop Improvement and Germplasm Enhancement, Gansu Agricultural University, Lanzhou 730070, China; ^2^Plant & Food Research Mt Albert, Private Bag 92169, Auckland Mail Centre, Auckland 1142, New Zealand; ^3^College of Life Science and Technology, Gansu Agricultural University, Lanzhou 730070, China; ^4^College of Horticulture, Gansu Agricultural University, Lanzhou 730070, China; ^5^College of Food Science and Engineering, Gansu Agricultural University, Lanzhou 730070, China; ^6^School of Biological Sciences, University of Auckland, Private Bag 92019, Auckland 1142, New Zealand

**Keywords:** Anthocyanin, cofactors, diversification, interaction, MYB transcription factors, potato.

## Abstract

In potato (*Solanum tuberosum* L.), R2R3 MYBs are involved in the regulation of anthocyanin biosynthesis. We examined sequences of these MYBs in cultivated potatoes, which are more complex than diploid potato due to ploidy and heterozygosity. We found amino acid variants in the C-terminus of the MYB StAN1, termed R0, R1, and R3, due to the presence of a repeated 10-amino acid motif. These variant MYBs showed some expression in both white and pigmented tubers. We found several new alleles or gene family members of R2R3 MYBs, *StMYBA1* and *StMYB113*, which were also expressed in white potato tubers. From functional analysis in tobacco, we showed that the presence of a C-terminal 10-amino acid motif is optimal for activating anthocyanin accumulation. Engineering a motif back into a MYB lacking this sequence enhanced its activating ability. Versions of *StMYBA1* and *StMYB113* can also activate anthocyanin accumulation in tobacco leaves, with the exception of *StMYB113-3*, which has a partial R2R3 domain. We isolated five family members of potato *StbHLH1*, and one *StJAF13*, to test their ability to interact with MYB variants. The results showed that two alleles of *StbHLH1* from white skin and red skin are non-functional, while three other *StbHLH1*s have different co-regulating abilities, and need to be activated by StJAF13. Combined with expression analysis in potato tuber, results suggest that *StbHLH1* and *StJAF13* are key co-regulators of anthocyanin biosynthesis, while the transcripts of MYB variants *StAN1*, *StMYBA1*, and *StMYB113* are well expressed, even in the absence of pigmentation.

## Highlight

We report evidence of the diversification and interaction of anthocyanin-related MYB activators and basic helix-loop-helix cofactors, which regulate anthocyanin biosynthesis in the potato tuber.

## Introduction

Anthocyanins, the largest group of plant flavonoids, are the main pigments responsible for the red-blue colour of many plant species (Grotewold, 2006; Holton and Cornish, 1995; Petroni and Tonelli, 2011). Besides attracting pollinators and aiding in seed dispersal in flowers and fruits, anthocyanins have key roles in protection against UV radiation and cold temperatures (Christie *et al.*, 1994; Sarma and Sharma, 1999), and response to drought stress (André *et al.*, 2009; Castellarin *et al.*, 2007). Moreover, anthocyanins have potential health benefits in humans, such as protection against some cancers and neuronal and cardiovascular diseases (Crowe *et al.*, 2011; He and Giusti, 2010; Mink *et al.*, 2007).

The cytosol-located anthocyanin biosynthetic pathway enzymes are encoded by a series of well-described genes (Grotewold, 2006; Holton and Cornish, 1995; Payyavula *et al.*, 2013). After biosynthesis, anthocyanins are transported to vacuoles or cell walls (Koes *et al.*, 2005). Expression of the pathway genes is controlled by a complex of MYB transcription factors (TFs), basic helix-loop-helix (bHLH) TFs, and WD-repeat proteins, the MYB-bHLH-WD40 ‘MBW’ complex (Baudry *et al.*, 2004; Patra *et al.*, 2013). The MYB superfamily constitutes one of the most abundant groups of TFs described in plants. MYB proteins have two distinct regions, an N-terminal conserved MYB DNA-binding domain, which is approximately 52 amino acid residues in length, and a diverse C-terminal modulator region that is responsible for the regulatory activity of the protein. Based on the number of highly conserved imperfect repeats in the MYB domain, the MYB family can be divided into four classes, 1R-, R2R3-, 3R-, and 4R-MYB proteins (Dubos *et al.*, 2010; Rosinski and Atchley, 1998). Among these MYB TFs, R2R3 MYBs constitute the largest TF gene family in plants, with 126 R2R3 MYB genes identified in Arabidopsis and divided into 22 subgroups on the basis of conserved motifs (Stracke *et al.*, 2001). The R2R3 MYB family plays an important role in regulating the expression of catalytic enzymes, including the anthocyanin pathway (Allan *et al.*, 2008). Many R2R3 MYB regulators have been demonstrated to be transcriptional activators of the anthocyanin biosynthetic pathway from many species, such as Arabidopsis (Borevitz *et al.*, 2000; Gonzalez *et al.*, 2008), petunia (Quattrocchio *et al.*, 1999), tomato (Mathews *et al.*, 2003), grapevine (Deluc *et al.*, 2008; Kobayashi *et al.*, 2005; Walker *et al.*, 2007), maize (Paz-Ares *et al.*, 1987), pepper (Borovsky *et al.*, 2004), potato StAN1 (Jung *et al.*, 2005, 2009; Zhang *et al.*, 2009b), sweet potato (Chu *et al.*, 2013), and apple (Ban *et al.*, 2007; Espley *et al.*, 2007; Takos *et al.*, 2006). In addition to the transcriptional activators, several MYB TFs have been identified as repressors of the anthocyanin biosynthetic pathway from several species, for example, strawberry (Aharoni *et al.*, 2001), snapdragon (Tamagnone *et al.*, 1998), apple (Lin-Wang *et al.*, 2011), grapevine (Cavallini *et al.*, 2015), Arabidopsis (Jin *et al.*, 2000), petunia (Albert *et al.*, 2011), and ginkgo (Xu *et al.*, 2014), as well as a single MYB-repeat AtMYBL2 and At CPC (Dubos *et al.*, 2008; Matsui *et al.*, 2008; Zhu *et al.*, 2009).

Another crucial TF involved in anthocyanin biosynthesis is the bHLH protein. The first studied bHLH TF regulating the anthocyanin pathway was Lc from maize, which was shown to cooperate with the MYB factor C1 (Ludwig *et al.*, 1989); bHLH TFs were subsequently studied in other plants, such as AtbHLH42 (AtTT8) in Arabidopsis, AN1/JAF13 bHLHs in petunia, MdbHLH3 in apple, and VvMYC1 and VvMYCA1 in grapevine (Chandler *et al.*, 1989; Espley *et al.*, 2007; Goodrich *et al.*, 1992; Hichri *et al.*, 2010; Matus *et al.*, 2010). The bHLH TF is essential for the activity of the R2R3 MYB partner, as they interact with each other to form transcriptional complexes with the promoters of biosynthetic genes. For example, the R2R3 MYB C1 protein in maize (*Zea mays*) interacts with a bHLH TF (either of the genes termed *B* or *R*) to activate the promoter of dihydroflavonol reductase (*DFR*) (Sainz *et al.*, 1997). In contrast, maize P1 MYB activates some flavonoid genes in the absence of a bHLH (Grotewold *et al.*, 2000).

The variation of colour intensity, or the location of pigmentation, has been attributed to mutations in the genes or promoters of TFs. A retrotransposon-induced mutation in grapevine (*Vitis vinifera*) in the promoter region of *MYBA1* leads to a loss of anthocyanin accumulation in berry skin (Kobayashi *et al.*, 2004). Multiple repeats of a promoter segment causes TF autoregulation in red-fleshed apples (Espley *et al.*, 2009), while a motif of two 228bp fragments forming tandem repeats in the promoter region of the MYB TF *RLC1* causes red leaf coloration in cotton under light (Gao *et al.*, 2013). The Rc mutation in rice (*Oryza sativa*) accounts for 97% of white pericarp varieties (Sweeney *et al.*, 2007) and was shown to be a 14bp deletion in the bHLH *Rc* gene.

Potato (*Solanum tuberosum*) is a major staple food and the fourth largest crop grown worldwide. It has been found that pigmented potato cultivars are a rich source of anthocyanins, in particular acylated derivatives (Fossen and Andersen, 2000; Rodriguez-Saona *et al.*, 1999). The concentration of anthocyanins in purple-fleshed tubers is similar to that of the highest anthocyanin-producing crops, such as blueberries, blackberries, cranberries, or grapes (Puértolas *et al.*, 2013). Potato peel shows a higher anthocyanin content and antioxidant activity than the corresponding flesh (Lewis *et al.*, 1998). Anthocyanin synthesis in the tuber periderm of tetraploid potato is controlled by three loci, *D*, *P*, and *R*, which have been localized to chromosomes 11, 2, and 10, respectively. *P* and *R* were found to be genes encoding the biosynthetic enzymes F3′5′H and DFR, whereas *D* encodes an R2R3 MYB named StAN1, which is similar to petunia AN2 (Jung et al., 2005, 2009; Zhang *et al.*, 2009b). StAN1 is not only a crucial regulator of anthocyanin biosynthesis in skin coloration of potato, but also a key regulator of other tuber phenylpropanoids and is regulated by sucrose (Payyavula *et al.*, 2013). An additional MYB with high homology to AN1 protein, StMYBA1, corresponds to the translated sequence of the published *StAN3* and is a possible *AN1* pseudogene (D’Amelia *et al.*, 2014; Jung *et al.*, 2009). StMYB113 is homologous to AtMYB113, which positively regulates phenylpropanoid metabolism in *Arabidopsis thaliana* (Borevitz *et al.*, 2000). StbHLH1 shows a strong association with phenylpropanoid expression in the potato tuber and may contribute to the anthocyanin accumulation in potato leaves, while StJAF13 acts as a putative AN1 co-regulator for anthocyanin gene expression in potato leaves (D’Amelia *et al.*, 2014; Payyavula *et al.*, 2013).

In this study, functional characterization of alleles or gene family members of the R2R3 MYBs *StAN1*, *StMYBA1*, and *StMYB113* from different cultivated potatoes was performed. The function of the different versions of *StAN1*, showing variable C-terminal repeats, was verified, while *StMYBA1* and *StMYB113* also showed activation activity. Furthermore, five alleles or gene family members of *StbHLH1*, and one *StJAF13*, were also isolated and their function in anthocyanin biosynthesis was studied. The results showed that two alleles of *StbHLH1* from white and red cultivars were non-functional, and other alleles from purple and red cultivars had different levels of co-regulating ability. In potato, real-time quantitative PCR (qPCR) analysis of skin and flesh of tubers suggested that a lack of expression of *StbHLH1* and *StJAF13* limits anthocyanin biosynthesis, while *StAN1*, *StMYBA1*, and *StMYB113* are expressed in both white and pigmented tissues. However, the majority of transcripts of *StAN1* are truncated in unpigmented tissues. In white potato tubers, failure to activate expression of *StbHLH1* and *StJAF13* may be due to differential processing of the *StAN1* transcripts and non-functional alleles of *StbHLH1*.

## Materials and methods

### Plant materials

**Fig. 1.**
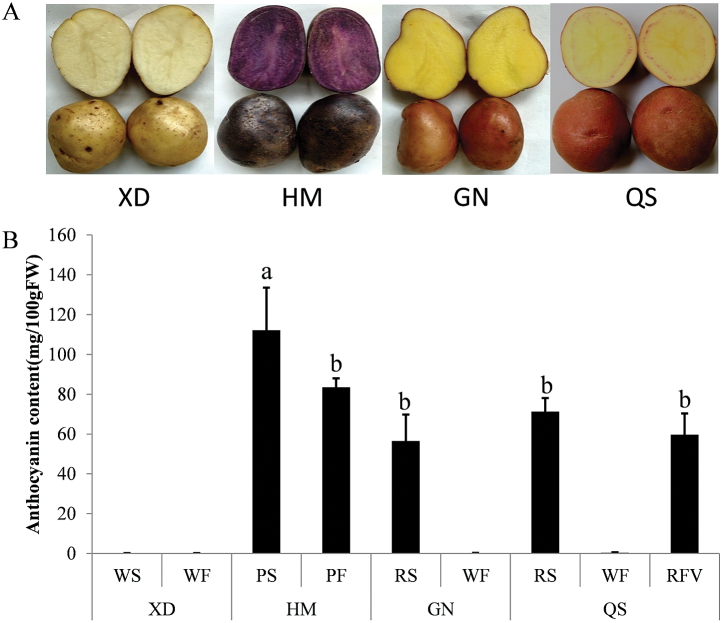
Characterization of the four genotypes used in this study. (A) Skin and flesh colour of Xin Daping (XD), Hei Meiren (HM), Gannongshu No.5 (GN) and Qingshu No.9 (QS). (B) Total anthocyanin content of skin, flesh, and red vascular ring of the four genotypes. The data represent the means±SE of three biological replicates. Statistical significance was determined by one-way ANOVA; significant differences between means [Least Significant Difference (LSD), *P*<0.05] are indicated where letters above the bars differ. WS, white skin; WF, white flesh; PS, purple skin; PF, purple flesh; RS, red skin; RFV, red vascular ring.

The white potato (*Solanum tuberosum* L.) cultivar ‘Xin Daping’ (XD; white skin and white flesh), purple potato cultivar ‘Hei Meiren’ (HM; purple skin and purple flesh), and red potato cultivars ‘Gannongshu No.5’ (GN; red skin and white flesh) and ‘Qinshu No.9’ (QS; red skin, white flesh and red vascular ring) (Fig. 1A) were grown in a greenhouse at Gansu Agricultural University, China. GN was cultivated by Gansu Agricultural University, HM and XD are local cultivars in Gansu province, and QS was cultivated by Qinghai Academy of Agriculture and Forestry Sciences of Qinghai Province. Five fresh tubers (diameter 4–5cm) were harvested and skin tissue was carefully separated from cortex tissue using a scalpel to minimize flesh contamination. The flesh tissue was isolated with at least 5mm distance from the skin to eliminate skin contamination. The red vascular ring was separated from surrounding white flesh tissue using a scalpel. The skin and flesh of these potatoes were frozen in liquid nitrogen and stored at –80 °C. 

*Nicotiana tabacum* and *Nicotiana benthamiana* were grown under glasshouse conditions in full potting mix, using natural light with daylight extension to 16h.

### Determination of anthocyanin content of potato skin and flesh

Anthocyanin content was determined by the pH differential spectrophotometry method described by Zhang et al (2009a). Anthocyanins were extracted from 1g samples in methanol/0.05 % HCl and absorbance of the extracts was measured by a spectrophotometer (UV-2550, Shimadzu, Japan) at 510 and 700nm. Absorbance (Abs) was calculated as Abs=(A_510 nm_–A_700 nm_)pH_1.0_–(A_510 nm_–A_700 nm_)pH_4.5_ with a molar extinction coefficient for cyanidin 3-glucoside of 26900 (Yang *et al.*, 2012; Zhang *et al.*, 2009a). Total anthocyanin content (TAC) was calculated using the following equation and expressed as milligrams of cyanidin 3-glucoside equivalents per 100g dry material.

$$\text{TAC}\left(\text{\%}\right)=\frac{\text{Abs}}{eL}\times \text{MW}\times D\times \frac{V}{G}\times 100$$

Where *e* is cyanidin 3-glucoside molar absorbance [26900mL (mmol·cm)^–1^], *L* is the cell path length (1cm), MW is the molecular weight of anthocyanin (449.2g mol^–1^), *D* is a dilution factor, *V* is the final volume (ml), and *G* is the mass of dry material (mg).

### DNA and RNA extraction

Total RNA of skin and flesh from the four potato cultivars, and from young leaves and roots of tobacco, were extracted using the PureLink Plant RNA Reagent Kit (Invitrogen, USA) according to the manufacturer’s instructions. The RNA was quantified by using a Nanodrop ND-1000 spectrophotometer (Nanodrop Technologies, USA) and quality was assayed on a 1% agarose gel. Removal of genomic DNA and first-strand cDNA synthesis were carried out using oligo(dT) (QuantiTect Reverse Transcription Kit, Qiagen). Genomic DNA was extracted from tuber tissue using the cetyl trimethyl ammonium bromide method of Murray and Thompson (1980).

### Gene cloning and sequence analysis

Full-length coding sequences of the alleles of the potato MYBs *StAN1*, *StMYBA1*, and *StMYB113*, and the potato bHLHs *StbHLH1* and *StJAF13*, were amplified from cDNA of skin and flesh of four cultivars using Platinum *Taq* DNA Polymerase High Fidelity (Invitrogen, USA). A truncated version of *StAN1*, *StAN1-R0T*, was amplified from cDNA of white skin. Full-length fragments were ligated into the binary vectors pSAK277 or pHEX2. Promoter sequences were isolated from the four potato cultivars and inserted into the pGreenII 0800-LUC vector (Hellens *et al.*, 2005). Details of all the cloning procedures are shown in the Supplementary Methods at *JXB* online and primers used are described in Supplementary Table S3.

The sequences of non-functional *StMYB113-3*, *StbHLH1-1*, and *StbHLH1-4* are described in Supplementary Table S4. Conserved cis-element motifs located in promoters were scanned by using the online software PLACE (http://bioinformatics.psb.ugent.be/webtools/plantcare/html/ and http://www.dna.affrc.go.jp/PLACE/). All constructs were individually electroporated into *Agrobacterium tumefaciens* GV3101 (MP90). The *NtAN1* RNAi and *NtJAF13* RNAi binary vector pTKO2 and the promoter of Arabidopsis *DFR* (TT3, AT5g42800) were from the New Zealand Institute for Plant & Food Research Ltd (Hellens *et al.*, 2005; Lin-Wang *et al.*, 2010).

### Phylogenetic analysis

Phylogenetic trees of MYB TFs and bHLH TFs were developed using MEGA6.0 (Tamura *et al.*, 2007). The evolutionary history was inferred using the minimum evolution phylogeny test and 1000 bootstrap replicates. The evolutionary distances were computed using the Poisson correction method with units of the number of amino acid substitutions per site.

### Transient assays of gene function

Transient assays, or dual luciferase assays, were performed in tobacco (*N. benthamiana* or *N. tabacum*) as previously reported (Espley *et al.*, 2009; Lin-Wang *et al.*, 2010). Details of all the transient assay procedures are shown in the Supplementary Methods.

In a separate colour assay, *NtAN1 RNAi* and *NtJAF13 RNAi* stably transformed tobacco plants were used (Montefiori *et al.*, 2015). Digital photographs of anthocyanin development in these patches were taken at 4 days post-infiltration.

### Transformation of tobacco

*StAN1-R0*, *StAN1-R1*, and *StAN1-R3* were transformed in tobacco leaves (*N. tabacum*) using the leaf disc method (Horsch *et al.*, 1985). After inoculation of leaf discs on medium with kanamycin and Timentin (ticarcillin disodium and clavulanate potassium), resistant shoots were regenerated from the cut surface of the explants. These shoots were separated from the explants and roots were induced. For each construct, 12 transgenic lines were obtained. Transgenic plants were identified by kanamycin selection and by qPCR analysis.

### HPLC measurement of tobacco leaves and roots

For the stably transformed tobacco plants, three tobacco leaves were taken and pooled together from each of five transgenic lines, and roots were taken from each of three transgenic lines for each construct, and empty vector pSAK277 was used as a negative control. For transient assays, three patches of tobacco leaf were pooled together for each treatment, with three biological replicates, and GUS was used as a negative control. Freeze-dried tissue was pulverized and resuspended in methanol (with 0.1% HCl) at a ratio of 5ml solvent to 1g of original fresh weight (FW). The mixture of powdered sample and solvent was extracted at room temperature for 3h in the dark, and then centrifuged at 3500rpm for 10min. The supernatant was diluted 20-fold with 20% methanol. A 400 μl aliquot of the diluted supernatant was used for anthocyanin HPLC measurement of tobacco leaves (McGhie *et al.*, 2005).

### qPCR

Real-time qPCR DNA amplification and analysis was carried out using the LightCycler 480 Real-Time PCR System (Roche), with LightCycler 480 software version 1.5. The LightCycler 480 SYBR Green I Master Mix (Roche) was used. qPCR conditions were 5min at 95 °C, followed by 40 cycles of 5s at 95 °C, 5s at 60 °C, and 10s at 72 °C, followed by 65–95 °C melting curve detection. The qPCR efficiency of each gene was obtained by analysing the standard curve of a cDNA serial dilution of that gene. Relative abundance was calculated with the ΔC_T_ method using actin (X55752) and elongation factor-1 (AB061263) of potato, and actin (EU938079) and elongation factor-1 (D63396) of tobacco for template normalization. The primers are listed in Supplementary Table S3.

### R repeat function verification

A synthesized cDNA version of *StAN1-R0* (with a R motif inserted), termed *StAN1-R0M*, in pUC57 cloning vector (GenScript), was used for R repeat function verification. The R repeat was inserted in the same position in *StR0M* as it is found in *StR1* (amino acids 132–143), then cloned into the pSAK277 expression vector. This construct was infiltrated into *N. benthamiana* and *N. tabacum* leaves, as described previously, in the presence of the *prom-3-StDFR* promoter to test the R repeat function.

### Statistical analysis

For qPCR analysis, data are presented as means (±SE) of four biological replicates. For transient transformation promoter activation assays, data are presented as means (±SE) of four biological replicates. For the analysis of anthocyanins, data are presented as means (±SE) of three biological replicates. Statistical significance was determined by one-way ANOVA.

## Results

### Tuber anthocyanin content in four potato genotypes

Four genotypes, HM (purple skin and purple flesh), GN (red skin and white flesh), QS (red skin, white flesh and red vascular ring), and XD (white skin and white flesh), were analysed (Fig. 1A). Anthocyanins were detectable only in red or purple skin or flesh, with the highest concentration in the purple skin (112.2±21.29mg 100g^–1^ FW). The concentrations of anthocyanins in the purple flesh (83.5±4.49mg 100g^–1^ FW), red skin and flesh (71.3±6.70mg 100g^–1^ FW and 56.54±13.25mg 100g^–1^ FW, respectively), and red vascular ring (59.68±10.62mg 100g^–1^ FW) were not significantly different (Fig. 1B).

### Analysis of *StAN1*, *StMYBA1*, and *StMYB113* gene sequences in differentially pigmented cultivars

**Fig. 2.**
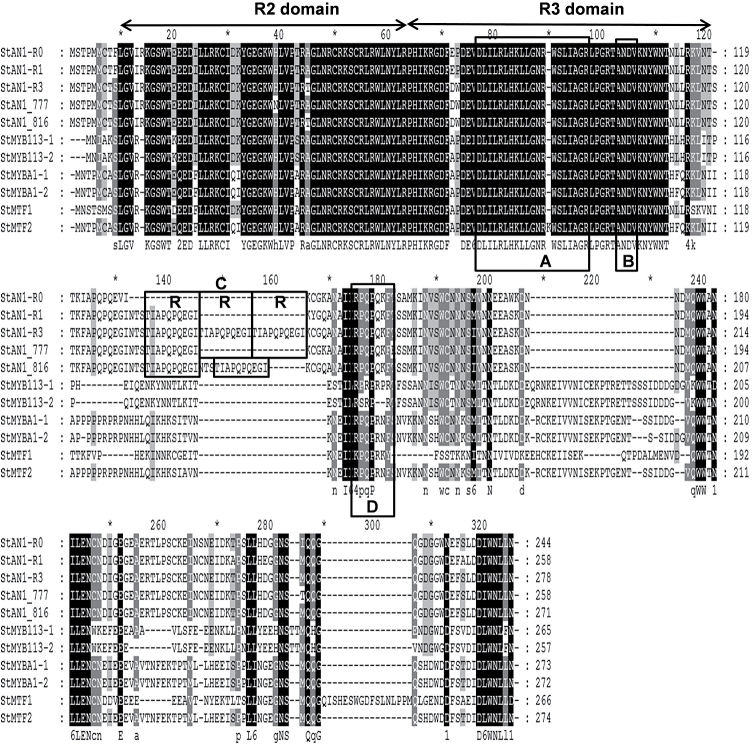
Protein sequence alignment of anthocyanin MYB regulators from potato. The R2 and R3 repeat domains are indicated by arrows. Box (A) indicates the conserved region of the bHLH interacting motif ([DE]Lx2[RK]x3Lx6Lx3R). Box (B) indicates a conserved motif [A/S/G]NDV in the R2R3 domain for dicot anthocyanin-promoting MYBs. Box (C) indicates the perfect repeat TIAPQPQEGI. Box (D) indicates a C-terminal-conserved motif [R/K]Px[P/A/R]xx[F/Y] for anthocyanin-regulating MYBs (Hichri *et al.*, 2010). NCBI protein accession numbers: StAN1-R0, AKA95391; StAN1-R1, AKA95392; StAN-R3, AKA95392; StAN1_777, AAX53089; StAN1_816, AAX53087; StMYB113-1, ALA13583; StMYB113-2, ALA13584; StMYBA1-1, ALA13581; StMYBA1-2, ALA13582; StMTF1, ABY40370; StMTF2, ABY40371.

PCR amplification and genotyping were used to determine the sequences of *StAN1* variants in differentially pigmented tissues of potato cultivars. Full-length coding sequences of three variants, termed *StAN1-R0*, *StAN1-R1*, and *StAN1-R3*, were amplified from cDNA of tuber skin and flesh of the four cultivars (Fig. 1, Table 1). Distinguishing these variants were three perfect duplications of 30 nucleotides (CTATTGCTCCTCAACCACAAGAAGGAATTA) termed R (coding for 10 amino acids: TIAPQPQEGI) in the third exon of *StAN1-R3* (Fig. 2). A truncated version of *StAN1-R0*, termed *StAN1-R0T*, was amplified from *StAN1-R0* at positions 1–302bp according to our previous RNA-seq result (Liu *et al.*, 2015) (Table 1).

**Table 1.** Presence of different transcription factor genes and alleles in tetraploid potatoes used in this study.

	Cultivar and tissue								
	XD		HM		GN		QS		
Transcript	WS	WF	PS	PF	RS	WF	RS	WF	RFV
*StAN1-R0*	√								
*StAN1-R1*		√	√	√		√√			
*StAN1-R3*	√√				√	√	√√	√√	√
*StAN1-R0T*	√								
*StMYBA1-1*	√	√	√	√	√	√	√	√	√
*StMYBA1-2*			√						
*StMYB113-1*	√		√						
*StMYB113-2*					√		√		
StMYB113-3*			√						
StbHLH1-1*	√								
*StbHLH1-2*	√		√	√					
*StbHLH1-3*			√	√					
StbHLH1-4*					√				
*StbHLH1-5*					√				
*StJAF13*			√		√				

WS, white skin; WF, white flesh; PS, purple skin; PF, purple flesh; RS, red skin; RFV, red vascular ring. * Premature stop codon in this transcript. √ Full-length transcript identified by PCR and sequencing. √√ Identified by qPCR melting curve analysis followed by sequencing of the qPCR fragment.

Melting curve analysis and sequencing of the qPCR fragments confirmed expression of two variants, *StAN1-R0* and *StAN1-R3*, in white skin, but just one variant, *StAN1-R1*, was expressed in white flesh of the white cultivar XD (Table 1). Only *StAN1-R1* was expressed in purple skin and purple flesh. In red cultivars, *StAN1-R3* was expressed in red skin, white flesh, and the red vascular ring, while *StAN1-R1* was also present in the white flesh of the red cultivar GN (Supplementary Fig. S1).

Two variants of *StMYBA1* were also cloned, termed *StMYBA1-1* (expressed in all tissues) and *StMYBA1-2* (isolated only from HM purple skin). Distinguishing the two variants were three nucleotides (CCT) at positions 370–372 in the third exon of *StMYBA1-1*. Three differentially expressed variants of *StMYB113* were isolated, termed *StMYBA113-1*, *StMYBA113-2*, and *StMYBA113-3* (Fig. 2, Table 1). A 130bp deletion in *StMYBA113-3* at nucleotide positions 125–254 resulted in a premature stop codon at amino acid position 9. Compared with *StMYBA113-1*, there are several deletions and amino acid changes in *StMYBA113-2*, as shown in Fig. 2.

*StAN1-R0*, *StAN1-R1*, *StAN1-R3*, *StMYBA1-1*, *StMYBA1-2*, *StMYB113-1*, and *StMYB113-2* encoded R2R3 MYB TFs that contain the highly conserved R2 and R3 MYB domains in the N-terminal region. In addition, they all contained other conserved motifs in the C-terminal region, including the [D/E]Lx2[R/K]x3Lx6Lx3R motif (Box A in Fig. 2) critical for interaction with R/B-like bHLH proteins (Zimmermann *et al.*, 2004) and the conserved ANDV motif (Box B in Fig. 2) identified from MYB regulators of the anthocyanin pathway in Rosaceae (Lin-Wang *et al.*, 2010). StAN1-R0, StAN1-R1, StAN1-R3, StMYBA1-1, and StMYBA1-2 contained the motif [R/K]Px[P/A/R]xx[F/Y] (Box D in Fig. 2), which is highly conserved in the anthocyanin-activating MYBs of some plant species (Lin-Wang *et al.*, 2010), while in StMYB113-1 and StMYB113-2 this motif is [R/K] [P/S]x[P/A/R]xx[F/Y/R].

**Fig. 3.**
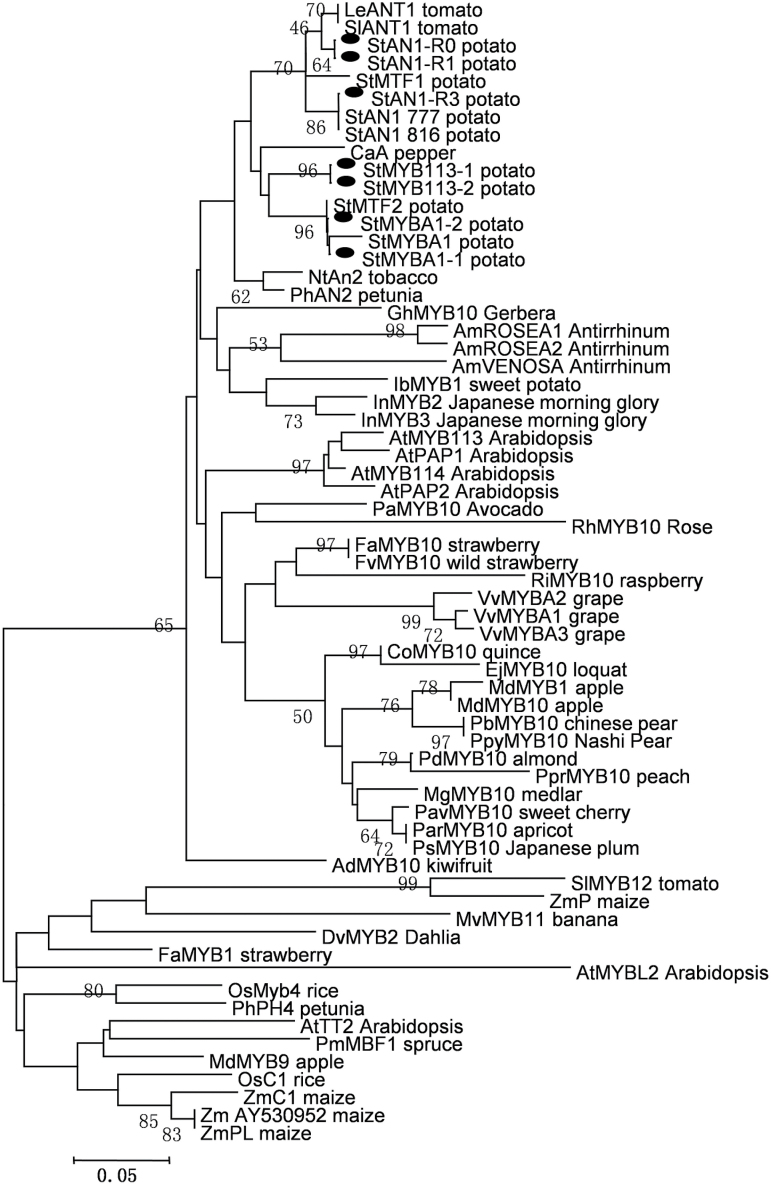
Phylogenetic relationship analysis of potato MYBs and known anthocyanin MYB regulators from other species. Sequences were aligned using Geneious v.6.1.6 (Drummond *et al.*, 2011) with a cost matrix of 65%, a gap open penalty of 12, and a gap extension penalty of 3. Phylogenetic and molecular evolutionary analysis was conducted using MEGA version 6.0. The evolutionary history was inferred using the neighbour-joining method and 1000 bootstrap replicates; bootstrap values less than 50 are not shown. The predicated proteins of StAN1-R0, StAN1-R1, StAN1-R3, StMYBA1-1, StMYBA1-2, StMYB113-1, and StMYB113-2 are indicated by black oval dots.

Newly identified *StAN1*, *StMYBA1*, and *StMYB113* sequences were highly homologous to previously identified MYBs in diploid (Jung *et al.*, 2009) and tetraploid potato: StAN1-777, StAN1-816 (AY841129 and AY841127), MTF1 and StMTF1 (EU310399), and StMTF2 (EU310400). Phylogenetic analysis (Supplementary Fig. S2) showed that StAN1-R0, StAN1-R1, StAN1-R3, StMYBA1-1, StMYBA1-2, StMYB113-1, and StMYB113-2 clustered with published regulators of anthocyanin biosynthesis such as PAP1, as well as other anthocyanin-promoting MYBs from dicot species. Monocot sequences, such as C1 of rice and P of maize, as well as the gymnosperm *Picea mariana* MBF1, clustered outside this group. Furthermore, the MYB proteins clustered according to their taxonomic relationships in Solanaceae and other anthocyanin-promoting MYBs from other species (Fig. 3). StAN1-R0, StAN1-R1, and StAN1-R3 were closely associated with StAN1-777, StAN1-816, and StMFT1. StMYBA1s were closely clustered with the StMFT2 and StMYB113s, as well as MYBs of other solanaceous species, SlANT1, LeANT1, PhAN2, NtAN2, and CaA, known to regulate anthocyanins.

### qPCR analysis of *StAN1*, *StMYBA1*, and *StMYB113* in four potato genotypes

**Fig. 4.**
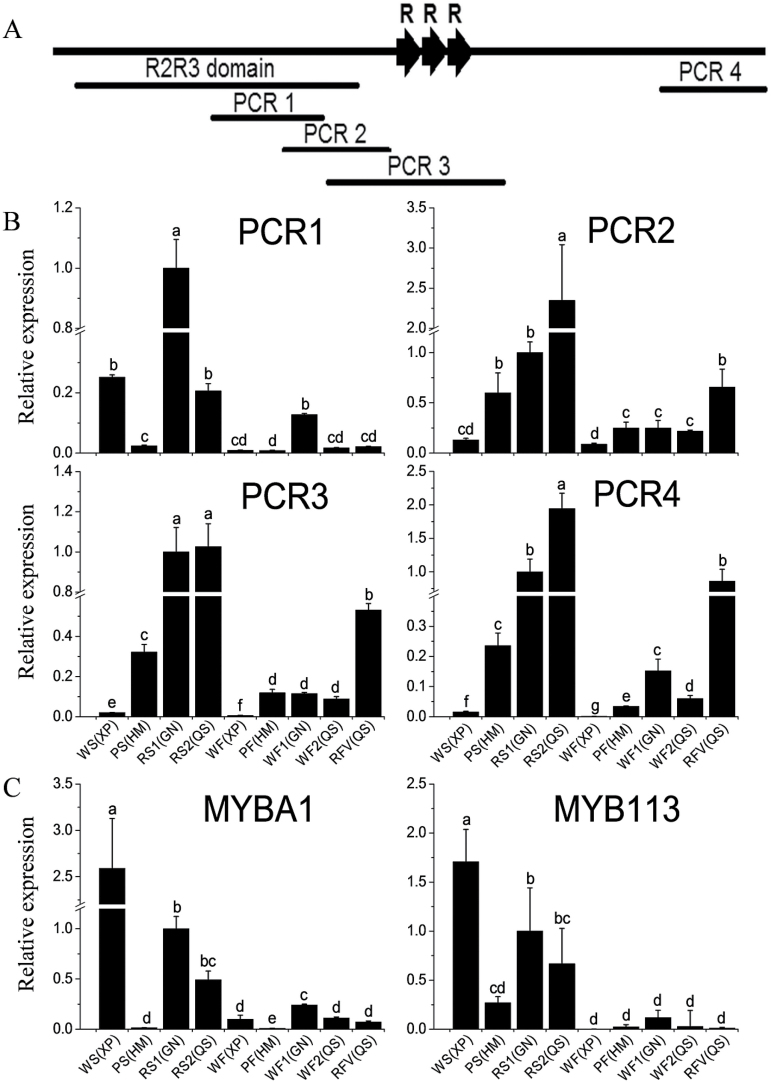
Expression analysis of StAN1s, StMYBA1s, and StMYB113s in four potato cultivars. (A) Schematic of the *StAN1* gene. PCR 1–4 represent the different regions. (B) qPCR expression of the different regions of *StAN1* in skin, flesh, and red vascular ring of the four genotypes, XD, HM, GN, and QS. Red skin of GN was set as a calibrator. (C) qPCR expression of *StMYBA1s* and *StMYB113s* in skin, flesh and red vascular ring of the four genotypes. Red skin of GN was set as a calibrator. The data represent the means±SE of three biological replicates. Statistical significance was determined by one-way ANOVA; significant differences between means (LSD, *P*<0.05) are indicated where letters above the bars differ. WS, white skin; PS, purple skin; RS, red skin; WF, white flesh; PF, purple flesh; RVF, red vascular ring.

To investigate the expression profiles of the different variants of *StAN1* in potato tubers, four pairs of qPCR primers were designed to different regions of *StAN1* (Fig. 4A). These were designated as PCR 1–4. PCR 1 was in the R2R3 domain, PCR 2 was before the repeat region including part of R2R3 domain, PCR 3 flanked the repeat region, and PCR 4 was in the C-terminus (Fig. 4A). These were used to test the different variants present in skin and flesh according to Tm value and sequence results.

Surprisingly, in the white skin of the white cultivar XD, *StAN1* expression in the region encoding the R2R3 domain (PCR 1) was much higher than in the purple skin of the purple cultivar (Fig. 4B). However, reduced expression was detected using primers for PCR 2 and PCR 3 regions, and there was very low expression in the white cultivar if the primers were targeted to the 3′-end of the cDNA (PCR 4). In contrast, only a small drop in amplification between PCR 1 and PCR 2, and consistent expression between PCR 2 and 3, was observed for the pigmented tissue and white flesh of the pigmented cultivars HM, GN, and QS. Amplification with primers targeted to the 3′-end of the cDNA was consistently detected in pigmented tissues, particularly in red cultivars. This is in agreement with our previous RNA-seq analysis (Liu *et al.*, 2015) where, in a white skin library, 1187 reads mapped to the R2R3 domain and only 13 reads mapped to the C-terminus. In a purple skin library, 579 total reads mapped evenly to both the R2R3 domain (297 reads) and the C-terminus (282 reads). These results suggest that most of the *StAN1* transcripts in the cultivar with white skin and white flesh lack the 3′-end, which encodes the C-terminus. Potentially, these truncated *StAN1* transcripts are unable to regulate anthocyanin biosynthesis.

In purple flesh, *StAN1* expression is moderate, but a full-length coding sequence is transcribed. However, there was little difference in expression between white flesh of red cultivars and the purple flesh, suggesting that, in addition to the expression level, the function of the three *StAN1* variants may differ. The expression profiles of *StMYBA1* and *StMYB113* were similar (Fig. 4C). Both had higher expression in the white skin of the white cultivar than in the purple skin of the purple cultivar. Their expression levels in the flesh of the four genotypes were lower than that in skin, and there was almost no expression of *StMYBA1* in purple flesh.

### Analysis of StbHLH TFs in differentially pigmented cultivars

Full-length coding sequences of five alleles or variants of the potato *StbHLH1* and one version of *StJAF13* were amplified from cDNA of tuber skin and flesh of the four cultivars (Table 1). These were termed *StbHLH1-1* (from skin of the white cultivar XD), *StbHLH1-2* (from skin of the white cultivar XD, and purple skin and purple flesh of HM), *StbHLH1-3* (from purple skin and purple flesh of cultivar HM), *StbHLH1-4*, and *StbHLH1-5* (from red skin of GN). *StJAF13* was isolated from purple skin of cultivar HM and red skin of GN. Supplementary Table S1 summarizes potential mutations found in the five *StbHLH1* variants with respect to published *StbHLH1* sequence. We found that the coding sequences of *StbHLH1-1* and *StbHLH1-2* were similar, except that *StbHLH1-1* showed a G nucleotide deletion at nucleotide position 1589, which resulted in a premature stop codon. The coding sequence of *StbHLH1-4* had an insertion of 50 nucleotides at nucleotide position 478–527, which also resulted in a premature stop codon. *StbHLH1-2* was the same as the published *StbHLH1*. *StbHLH1-3* had two deletions of 6 and 15 nucleotides, and six single nucleotide polymorphism (SNP) differences. *StbHLH1-5* showed a deletion of 24 nucleotides and 34 SNP differences (Supplementary Fig. S3, Table S1). There was 80.6, 100, 96.8, 35.2, and 96.8% identity between the five *StbHLH1* variants and published *StbHLH1*, respectively. *StJAF13* shared 99.8% identity with published *StJAF13*. Phylogenetic analysis showed that the five variants of *StbHLH* grouped with tobacco *An1a* and *An1b*, and petunia *An1* (Supplementary Fig. S4). *StJAF13* is grouped with tomato *GLABRA3*-like, tobacco *JAF13a* and *JAF13b*, and petunia *JAF13*.

### qPCR analysis of *StbHLH*s in four potato genotypes

**Fig. 5.**
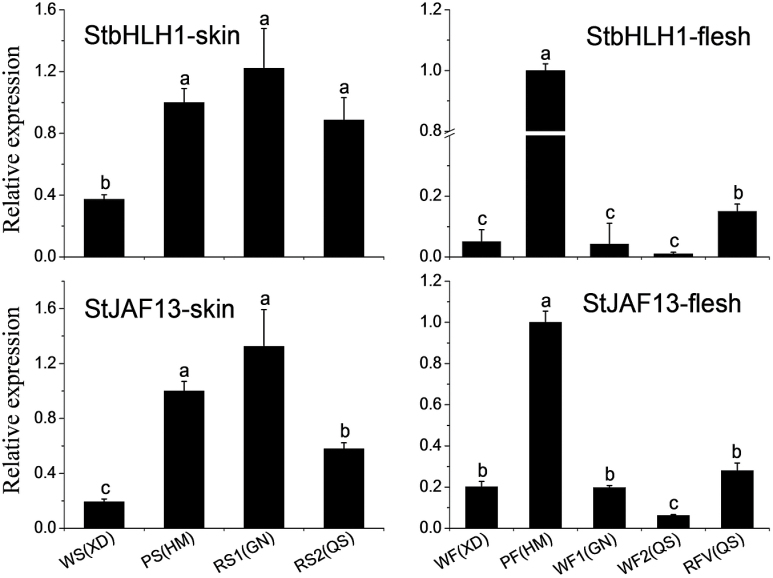
Quantitative analysis of transcript levels of *StbHLH1* and *StJAF13* in skin, flesh and red vascular ring of four genotypes. Purple skin and purple flesh of HM were set as calibrators. The data represent the means±SE of three biological replicates. Statistical significance was determined by one-way ANOVA; significant differences between means (LSD, *P*<0.05) are indicated where letters above the bars differ. WS, white skin; PS, purple skin; RS, red skin; WF, white flesh; PF, purple flesh; RVF, red vascular ring.

It has been previously shown in Solanaceous species that there is a hierarchy of bHLHs partnering with anthocyanin-activating MYBs (Montefiori *et al.*, 2015). We examined the expression of *StbHLH1* (Payyavula *et al.*, 2013) and *StJAF13* (D’Amelia *et al.*, 2014) in potato tubers. qPCR primers were designed based on the conserved region of the five alleles of *bHLH1*, allowing all versions to be detected. Both *bHLH*s showed similar expression profiles, with higher levels detected in genotypes with pigmented skin and flesh (Fig. 5, Table 1). The expression level was less in white skin than in pigmented skin. There was little expression of *StbHLH1* and *StJAF13* in white flesh of white and red-skinned cultivars, whereas they were both highly expressed in purple flesh. Expression was also elevated in the red vascular ring of the flesh in the QS cultivar (Fig. 5). Therefore, both *StbHLH1* and *StJAF13* correlate with anthocyanin biosynthesis in potato tuber skin and flesh.

### Functional assays of StAN1, StMYBA1 and StMYB113 in tobacco

In order to functionally test the different *MYB* genes and their variants, transient luciferase assays in *N. benthamiana* were used to measure transactivation of potato *DFR* and *F3′5′H* promoters. Sequencing of a ~2000bp region upstream of the ATG translation start codon of *StDFR* (chr02:40292119..40294109 PGSC, http://solanaceae.plantbiology.msu.edu/pgsc_download.shtml) (Potato Genome Sequencing Consortium, 2011) revealed substantial variation in the promoter. There were at least three alleles of *StDFR* in four genotypes, of which *prom-1-StDFR* and *prom-2-StDFR* were found in the white genotype XD, *prom-3-StDFR* was found in the purple and red cultivars HM and GN, and *prom-1-StDFR* and *prom-3-StDFR* were found in the other red cultivar QS. The *StF3′5′H* promoters of four genotypes were all identical. Numerous cis-acting regulatory elements were identified and the most abundant motifs were light-responsive elements such as G-Box, defence and stress-responsive elements (TC-rich repeats), and MYB binding sites (MYBCORE). A circadian element, methyljasmonate responsive element (CGTCA motif), Plant MYB binding site (MYBPLANT), and MYC recognition sequence (MYCATERD1) were present in all *DFR* promoters, but not in the *StF3′5′H* promoter (Supplementary Table S2).

**Fig. 6.**
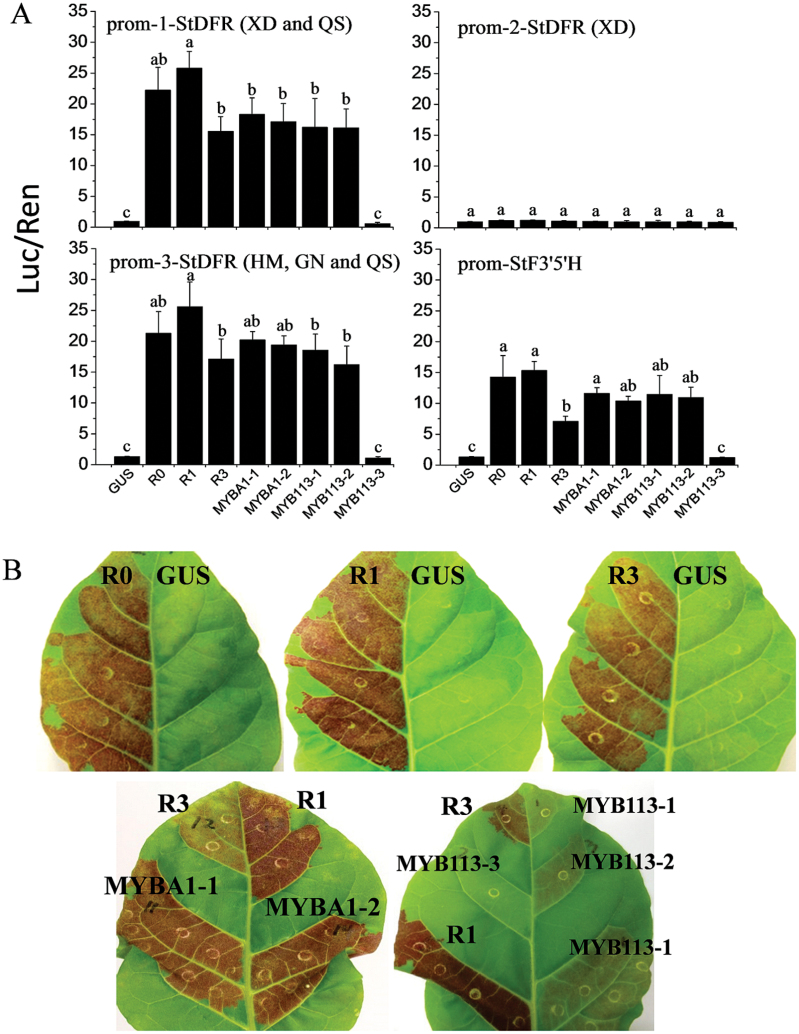
Transient assays of StAN1-R0, StAN1-R1, StAN1-R3, StMYBA1-1, StMYBA1-2, StMYB113-1, StMYB113-2, and StMYB113-3. (A) Activation of three alleles of potato DFR promoters and F3′5′H promoter. Error bars are the SE of three independent experiments with four replicate reactions. Statistical significance was determined by one-way ANOVA; significant differences between means (LSD, *P*<0.05) are indicated where letters above the bars differ. (B) Patches of anthocyanin production in tobacco leaves induced by infiltration with StAN1-R0, StAN1-R1, StAN1-R3, StMYBA1-1, StMYBA1-2, StMYB113-1, StMYB113-2, StMYB113-3, and GUS.

Full-length cDNAs of *StAN1-R0*, *StAN1-R1*, *StAN1-R3*, *StMYBA1-1*, *StMYBA1-2*, *StMYB113-1*, *StMYB113-2*, and *StMYB113-3* under control of the 35S promoter were co-infiltrated into *N. benthamiana* leaves with *prom-StDFRs-LUC* and *prom-StF3′5′H-LUC* in a second *Agrobacterium* strain (Hellens *et al.*, 2005). There was significant activation of *prom-1-StDFR* and *prom-3-StDFR* by seven of the eight potato MYBs, compared with the negative control (Fig. 6A). In contrast, *prom-2-StDFR*, cloned from the white cultivar, could not be activated by any of the eight MYBs. For the *prom-1-StDFR* and *prom-3-StDFR* promoters, activation by StAN1-R1 was higher than by StAN1-R3, StMYB113-1, and StMYB113-2. Activation of the *StF3′5′H* promoter by StAN1-R3 was significantly lower than by the other MYB TFs. StMYB113-3 appears to be non-functional as it lacks part of the R2R3 region.

The transient activation of an anthocyanic patch in tobacco was examined after infiltration with individual MYBs. No anthocyanin was observed with GUS or StMYB113-3. Only weak activation was detected upon infiltration of StMYB113-1 and StMYB113-2, and relatively strong activation was observed with the remaining MYBs (Fig. 6B). Significantly higher anthocyanin accumulation (as quantified by HPLC; Supplementary Fig. S5C) was observed upon infiltration of StAN1-R1 compared with StAN1-R3 (Fig. 6B), suggesting that the presence of a single R repeat provides optimal activation. This was confirmed using a mutant version of *StAN1-R0*, designated *StAN1-R0M*, in which an R motif is inserted into the same position as that found in *StAN1-R1.* Subsequent *prom-1-StDFR* transactivation assays combined with quantification of anthocyanin accumulation demonstrated a 1.22-fold increased activation capacity over StAN1-R0 (Supplementary Fig. S5). The truncated version of StAN1-R0, StAN1-R0T, did not induce any anthocyanin or significantly inhibit the activity of full-length StAN1-R0, R1, or R3 (Supplementary Fig. S6). These results suggest that the R repeat is an important functional domain in StAN1, with the single R motif enhancing the ability of StAN1 to activate anthocyanin synthesis.

### StAN1-R0, StAN1-R1, and StAN1-R3 activate all the anthocyanin biosynthetic genes in leaves and roots of transformed tobacco lines

In order to further examine the efficiencies of StAN1-R0, StAN1-R1, and StAN1-R3 in inducing anthocyanin biosynthesis, stably transformed lines of tobacco were generated. Transformed plants were tested by genomic PCR to confirm the presence of each transgene, and by qPCR to determine the individual expression levels of *StAN1-R0*, *StAN1-R1*, and *StAN1-R3*. For each construct, five independent lines were selected for phenotypic analysis.

**Fig. 7.**
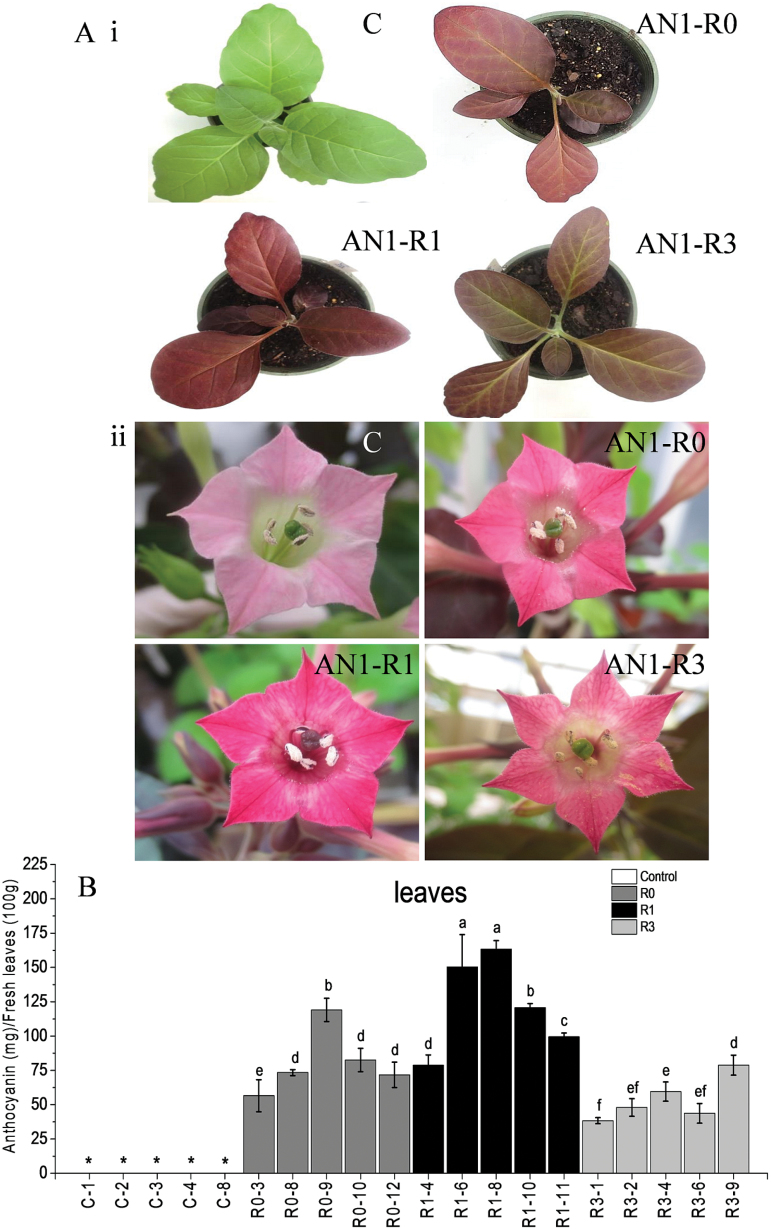
Phenotypes and anthocyanin content of transgenic tobacco plants transformed with empty vector, *StAN1-R0*, *StAN1-R1*, and *StAN1-R3*. (A) Visible reddening was seen in leaves (i) and flowers (ii) of plants transformed with *StAN1-R0*, *StAN1-R1*, and *StAN1-R3*. (B) Anthocyanin content from five independent transgenic lines of each construct showed the highest concentration in two out of the five *StAN1-R1* lines and the lowest concentration in three out of five *StAN1-R3* lines. C represents empty vector controls. Error bars are the SE for three replicate extracts per line. Statistical significance was determined by one-way ANOVA; significant differences between means (LSD, *P*<0.05) are indicated where letters above the bars differ. * indicates no detectable levels of anthocyanin content.

The lines overexpressing *StAN1-R1* accumulated significantly higher levels of anthocyanin in the leaves and flowers compared with those overexpressing *StAN1-R0* and *StAN1-R3* (Fig. 7A, B). The average foliar anthocyanin content in *StAN1-R1* transgenic lines was higher than in the *StAN1-R0* and *StAN1-R3* transgenic lines, with the highest anthocyanin content in the leaves of line 8 *StAN1-R1*, reaching 163.3±6.24mg 100g^–1^ FW (Fig. 7B).

**Fig. 8.**
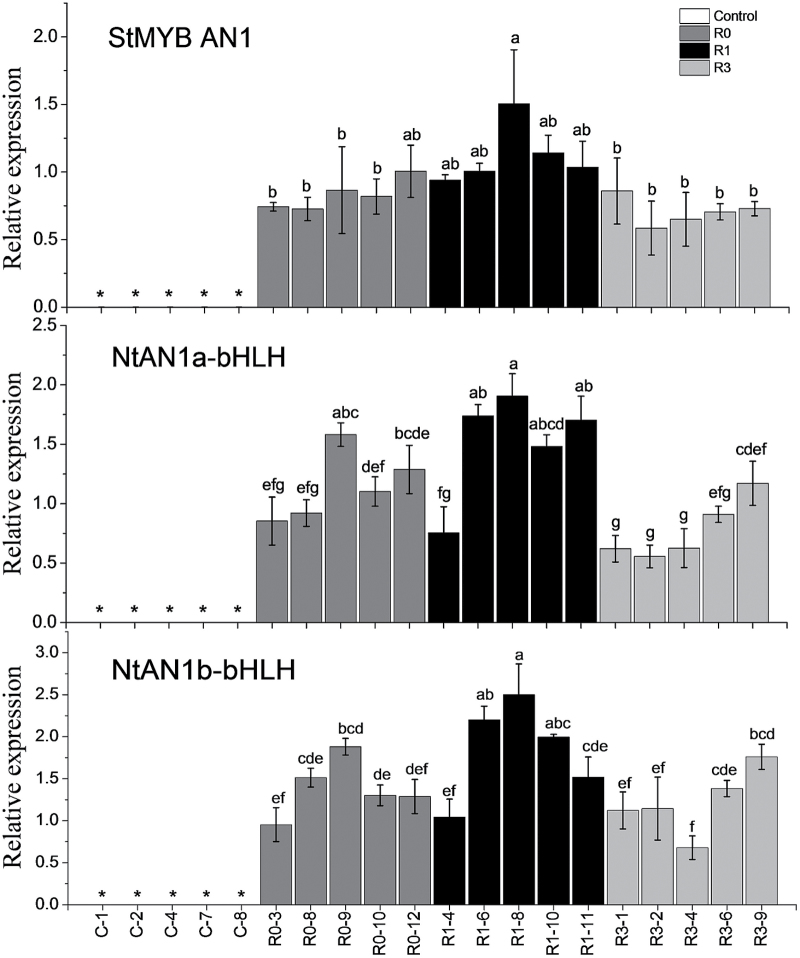
Quantitative analysis of transcript levels of *StMYB AN1*, *NtAN1a-bHLH*, and *NtAN1b-bHLH* in tobacco transgenic lines. The data represent the means±SE of three biological replicates. Statistical significance was determined by one-way ANOVA; significant differences between means (LSD, *P*<0.05) are indicated where letters above the bars differ. Genotypes denoted by * showed no detectable levels of expression.

The expression of *StAN1* and the endogenous tobacco bHLH TFs *NtAN1a* and *NtAN1b* were analysed in transgenic lines. The results showed that *StAN1-R0*, *StAN1-R1*, and *StAN1-R3* overexpression resulted in an induction of the endogenous bHLH TFs *NtAN1a* and *NtAN1b*. The induction of *NtAN1a* and *NtAN1b* was greatest in the leaves of *StAN1-R1* transgenic lines (Fig. 8). The tobacco MYB *NtAN2*, controlling tobacco anthocyanin production (Pattanaik *et al.*, 2010), was not induced by overexpression of the potato MYBs (not detectable by qPCR; not shown).

The transcript levels of several anthocyanin biosynthetic genes were also analysed, such as chalcone synthase (*NtCHS*), chalcone isomerase (*NtCHI*), flavonoid 3′-monooxygenase (*NtF3′H*), flavanone-3-beta-hydroxylase (*NtF3H*), and leucoanthocyanidin dioxygenase/anthocyanidin synthase (*NtLDOX/NtANS*). These were expressed at higher levels in *StAN1-R1* transgenic lines compared with *StAN1-R0* and *StAN1-R3* transgenic lines. The expression profile correlated with both the anthocyanin levels and endogenous bHLH TFs *NtAN1a* and *NtAN1b* (Supplementary Fig. S7).

### StAN1, StMYBA1, and StMYB113 partner with StbHLH1 and StJAF13 to regulate anthocyanin biosynthesis

As previously shown, StAN1-R0, StAN1-R1, StAN1-R3, StMYBA1-1, StMYBA1-2, StMYB113-1, and StMYB113-2 can all regulate anthocyanin biosynthesis in tobacco. In potato, *StAN1-R0*, *StAN1-R1*, and *StAN1-R3* are highly expressed in pigmented tissues, but they are also expressed in white skin and white flesh. qPCR and read mapping of RNA-seq suggests that the *StAN1* transcript is short in white tubers. However, *StMYBA1* and *StMYB113* are highly expressed in white skin of the white cultivar XD (Fig. 4C). This suggests that the presence or absence of StbHLH TFs may be crucial in regulating anthocyanin biosynthesis in potato. To further examine the interaction between *StbHLH1* and *StJAF13* with *StAN1*, *StMYBA1*, and *StMYB113*, transient assays were carried out in *N. tabacum* stably transformed with *RNAi NtAn1* (including *NtAn1a* and *NtAn1b*) and *RNAi StJAF13*. The aim of using these tobacco lines was to minimize the activity of the endogenous bHLHs.

**Fig. 9.**
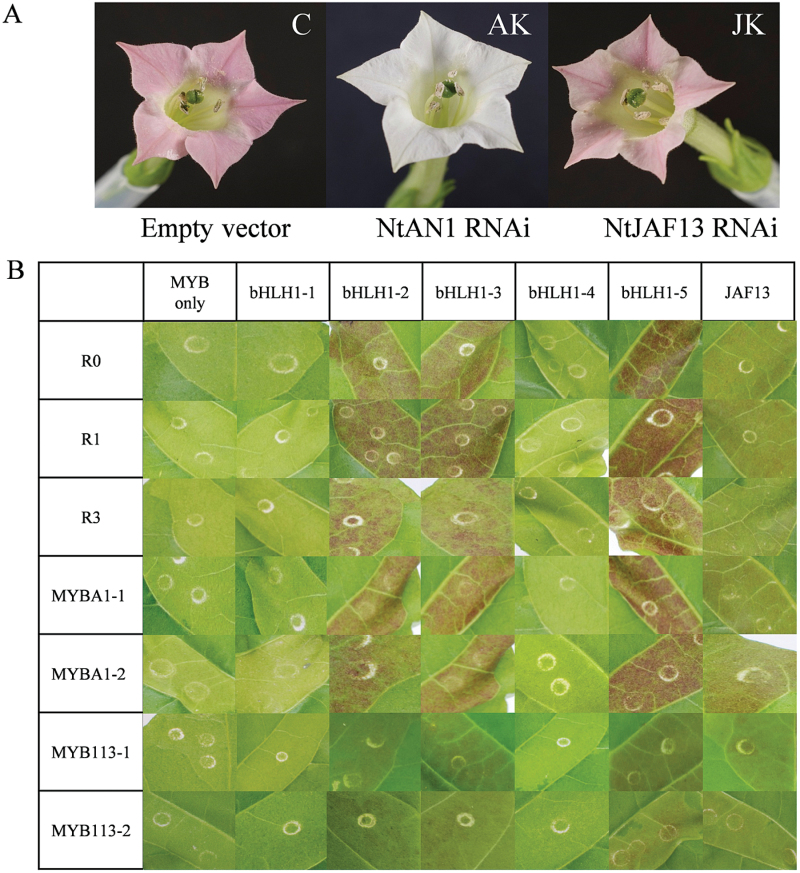
Transient activation of anthocyanic responses by *StAN1*, *StMYBA1*, and *StMYB113* combined with *StbHLH1* and with *StJAF13*. (A) Flowers of transgenic tobacco plants transformed with empty vector, *NtAn1 RNAi*, or *NtJAF13 RNAi*. (B) Patches of anthocyanin production in *NtAn1 RNAi* tobacco leaves infiltrated by MYBs alone or combined with five variants of *StbHLH1* and *StJAF13*.

As previously reported (Montefiori *et al.*, 2015), tobacco lines of *RNAi NtAn1* had white flowers and *RNAi NtJAF13* had pale pink flowers (Fig. 9A). The leaves of *RNAi NtAn1* plants were infiltrated with variants of StAN1 (StAN1-R0, StAN1-R1, and StAN1-R3), StMYBA1 (StMYBA1-1 and StMYBA1-2), or StMYB113 (StMYBA113-1 and StMYB113-2) alone, or combined with one of five variants of StbHLH1 (StbHLH1-1–StbHLH1-5) and one StJAF13. The *RNAi NtAn1* construct may have a negative effect on the potato *bHLH* transgenes. However, induction of anthocyanic patches indicates that this interference is minimal. When TFs were transiently transformed into *RNAi NtAn1* tobacco leaves, anthocyanin accumulation was observed in the treatments with *StAN1*, *StMYBA1*, and *StMYBA113* in the presence of *StbHLH1-2*, *StbHLH1-3*, *StbHLH1-5*, or *StJAF13*, but not with *StbHLH1-1* and *StbHLH1-4* or MYB TFs alone. The strongest anthocyanin accumulation was observed in leaves infiltrated with a MYB TF and *StbHLH1-5.* The weakest interaction appeared to be with the MYBs and *StJAF13* (Fig. 9B).

**Fig. 10.**
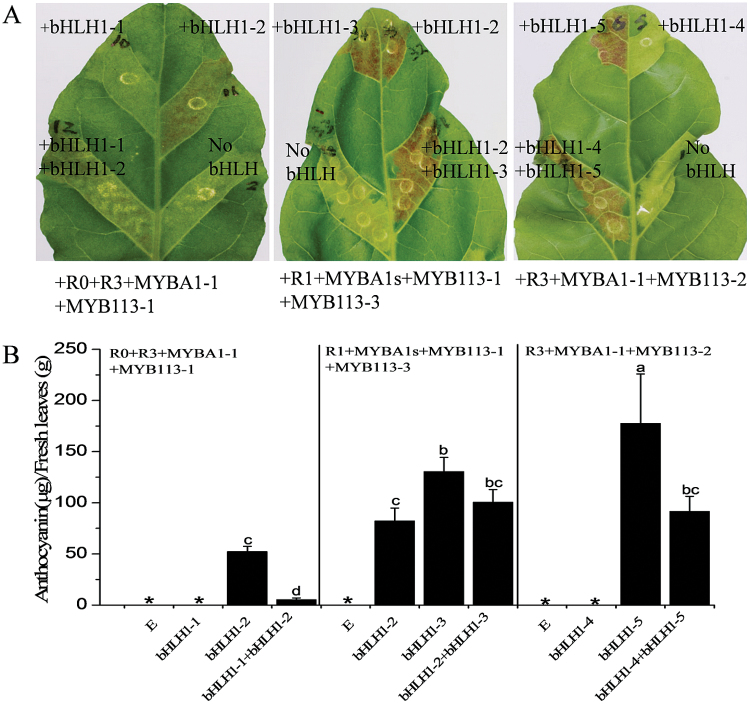
Transient activation of anthocyanic responses in tobacco by simulating transcription factors present in potato skin. (A) Patches of anthocyanin production in *NtAn1 RNAi* tobacco leaves induced by the combinations as shown. (B) Anthocyanin was extracted from patches of each combination. Error bars are the SE of three biological replicates. Statistical significance was determined by one-way ANOVA; significant differences between means (LSD, *P*<0.05) are indicated where letters above the bars differ. E represents empty vector control. Genotypes denoted by * showed no detectable levels of anthocyanin content.

To simulate the TFs present in the white potato skin, the MYBs *StAN1-R0*, *StAN1-R3*, *StMYBA1-1*, and *StMYB113-1* were mixed with *StbHLH1-1* and *StbHLH1-2* in a tobacco background lacking *NtAn1* (Fig. 10A). Anthocyanin production induced by the MYBs combined with *StbHLH1-1* and *StbHLH1-2* was significantly reduced compared with production induced by MYB TFs with *StbHLH1-2* alone (Fig. 10A, B). This raises the possibility that the non-functional *StbHLH1-1* and functional *StbHLH1-2* compete with each other, which limits anthocyanin accumulation.

In the purple skin of purple tubers, expression of the MYBs *StAN1-R1*, *StMYBA1-1*, *StMYBA1-2*, *StMYB113-1*, and *StMYB113-3*, and the bHLHs *StbHLH1-2* and *StbHLH1-3*, was evident. This combination induced high levels of anthocyanin accumulation in tobacco leaves. In the red skin of the red cultivar GN, *StAN1-R3*, *StMYBA1-1*, and *StMYB113-2*, and *StbHLH1-4* and *StbHLH1-5* transcripts were present, and this combination also induced anthocyanin accumulation in the *NtAn1 RNAi* tobacco leaves. The truncated non-functional *StbHLH1-4* did not prevent the accumulation of anthocyanin induced by the MYBs, yet had some inhibitory effect on the functional *StbHLH1-5* (Fig. 10A, B).

When the same combinations described above were transformed into *NtJAF13 RNAi* tobacco leaves, anthocyanin accumulation was prevented unless the MYBs were co-infiltrated with a functional bHLH (Supplementary Fig. S8). This supports observations that in the Solanaceae MYB TFs need to co-partner with *NtJAF13* to stimulate *NtAn1*, which then up-regulates the anthocyanin biosynthetic genes (Montefiori *et al.*, 2015). In the potato tuber, there was significantly lower expression of *StJAF13* in both white skin and white flesh (Fig. 5), suggesting that both *StJAF13* and *StbHLH1*s are important factors in controlling tuber anthocyanin biosynthesis.

## Discussion

For potato tuber skin and flesh, genetic analysis has revealed three major loci controlling pigmentation, *D*, *R*, and *P* (De Jong *et al.*, 2004). *StAN1*, coding for a R2R3 MYB, was shown to control potato skin colour, and *StMYBA1* was suggested as a possible *AN1* pseudogene (Jung *et al.*, 2009). In the present study, we characterized three variants of *StAN1* from four differentially pigmented genotypes and tested the importance of a repeated domain in the third exon of *StAN1*. We examined the function of *StMYBA1* and a novel *StMYB113*, both of which are highly expressed in white skin of white tubers. Results suggest that the StbHLH TFs are the limiting regulators in anthocyanin biosynthesis in the tuber, as MYBs (*StAN1-R0*, *StAN1-R1*, *StAN1-R3*, *StMYBA1*, and *StMYB113*) can be well expressed even in the absence of pigmentation.

### Three major variants of *StAN1* present in four different pigmented cultivars

In petunia, Schwinn *et al.* (2006) reported that striking effects on floral phenotype could be caused by small changes in MYB sequence. Recently, D’Amelia *et al.* (2014) reported that potato *AN1* displays high intraspecific sequence variability in both coding and non-coding sequences of *AN1* and that its expression in leaves is associated with high anthocyanin content. In studying the potato tuber, we confirmed this variability in *StAN1* and explored the function of many of the variants of the gene. Apart from several SNPs, the main difference between the variants was three perfect duplications of 30 nucleotides (termed R) in the third exon of *StAN1*. These indels would be consistent with the results of Jung *et al.* (2009), who found bands of different sizes of *StAN1* from different tetraploid potatoes. Their precise role is difficult to determine in this highly heterozygous crop. We therefore investigated the function of *StAN1-R0*, *StAN1-R1*, *StAN1-R3*, and the R repeat in more detail.

### The involvement of StAN1-R0, StAN1-R1, and StAN1-R3 in regulating anthocyanin biosynthesis

Since there are few other sequence differences between *StAN1-R0*, *StAN1-R1*, and *StAN1-R3*, it appears likely that this set of mutations occurred recently in the evolution or cultivation of potato. Our transient analysis revealed that *StAN1-R1*, harbouring one R-motif, was optimal for the regulation of anthocyanin levels. We further confirmed that the R-motif can enhance the ability of *StAN1* to regulate anthocyanin biosynthesis by inserting an R-motif into *StAN1-R0*.

The C-terminal variants of *StAN1* were further investigated using stable transformation of tobacco. Our results showed that heterologous expression of *StAN1-R0*, *StAN1-R1*, and *StAN1-R3* activated the anthocyanin biosynthetic pathway and induced anthocyanin pigmentation. Activated biosynthetic genes included the early biosynthetic genes *NtCHS*, *NtCHI*, *NtF3H*, and *NtF3′H*, and the late biosynthetic genes *NtDFR*, *NtANS*, and *NtUFGT*. In Arabidopsis, the expression of early and late biosynthetic genes appears to be controlled separately, by different TFs (Dubos *et al.*, 2010). In addition, the endogenous tobacco bHLH TFs *NtAN1a* and *NtAN1b* were highly induced. However, the endogenous MYB *NtAN2* was not induced by *StAN1-R0*, *StAN1-R1*, or *StAN1-R3*. This is in contrast to results found when bayberry *MrMYB1* was overexpressed in tobacco petals (Huang *et al.*, 2013).

### StbHLH1 and StJAF13 are limiting regulators of anthocyanin biosynthesis in potato tubers

The results of stable transformation showed that overexpression of *StAN1* leads to high expression of the *bHLH* genes *NtAN1a* and *NtAN1b* in tobacco. The expression profiles of the bHLH genes and biosynthetic genes are largely overlapping (Fig. 8, Supplementary Fig. S7). Conversely, in potato, *StAN1*, *StMYBA1*, and *StMYB113* can be highly expressed in white skin, but the expression levels of the bHLH partners appeared limiting. This suggests that bHLHs may be crucial in regulating anthocyanin biosynthesis in potato (De Jong *et al.*, 2004; Payyavula *et al.*, 2013). It was hypothesized by Jung *et al.* (2009) that for tuber flesh, the allelic configuration of different loci, like the *bHLH*s, may influence the phenotype when *AN1* is constitutively expressed. In Arabidopsis seeds, TT8/AtBHLH042 is involved in the control of the expression of the *DFR* and *BAN* genes (Nesi *et al.*, 2000). Appelhagen *et al.* (2011) identified EGL3 and TT8 as necessary regulators of anthocyanin accumulation in developing Arabidopsis seedlings. Butelli *et al.* (2012) observed that the RUBY MYB from orange (*Citrus sinensis*) promoted a stronger pigmentation of transformed tobacco plants when co-expressed with snapdragon bHLHs.

Based on qPCR in potato and transformation of tobacco, it appears that the production of anthocyanin in tubers is associated with two overlapping mechanisms. One requires a high level of expression of a full-length transcript of *StAN1*, and expression of *StbHLH1* and *StJAF13*, to activate anthocyanin biosynthesis. There is expression of a shortened *StAN1* transcript in the white skin of white tubers, while in white flesh of a red cultivar there is low expression of *StbHLH1* and *StJAF13*. Thus, there is no anthocyanin accumulation in these tissues despite the presence of high expression of *StMYBA1* and *StMYB113*. The other potential mechanism is linked to certain alleles of *StAN1* and *StbHLH1*, which influence anthocyanin biosynthesis. Different variants of *StAN1* have different anthocyanin-regulating abilities due to the presence of the R motif. Furthermore, amino acid substitutions in *StbHLH1-2*, *StbHLH1-3*, and *StbHLH1-5* cause alterations in the interaction with *StAN1*, and premature stop codons in *StbHLH1-1* and *StbHLH1-4* result in alleles which could be inhibitory in white-skinned tubers.

We also found that MYB TFs (*StAN1*, *StMYBA1*, and *StMYB113*) co-partner with *NtJAF13* to first activate the bHLH *NtAN1*, as previously shown (Montefiori *et al.*, 2015). The potato MYB TFs and the endogenous tobacco bHLHs then function together to up-regulate the anthocyanin biosynthetic genes. This is consistent with qPCR results of *StbHLH1* and *StJAF13* in potato, especially in flesh, where *StJAF13* expression patterns overlap with those of *StbHLH1*. D’Amelia *et al.* (2014) reported AN1/StJAF13 and AN1/StbHLH1 interactions in potato leaf; these are consistent with our results in tubers. It appears that *StJAF13* also has an important role in regulating anthocyanin biosynthesis in tubers.

In conclusion, these data show at least two important mechanisms controlling potato tuber pigmentation—an allelic diversity of variants of R2R3 MYBs and their interacting bHLH partners, as well as differential expression of key genes. Three *AN1* alleles (*StAN1-R0*, *StAN1-R1*, and *StAN1-R3*) have functionally important variations in the C-terminal domain, while *StMYBA1* and *StMYB113* are also potentially functional. We demonstrated that *StbHLH1* and *StJAF13* are also limiting factors in anthocyanin biosynthesis. Future work could focus on the alleles of *StAN1* and *StbHLH1* that potentially act as repressors of tuber pigmentation.

## Supplementary data

Supplementary Methods. Gene cloning and sequence analysis; transient assays of gene function.

Supplementary Table S1. Summary of missense mutations found with respect to *StbHLH1* (JX848660) sequence.

Supplementary Table S2. List of putative regulatory elements present in promoters of *StDFR* and *StF3′5′H*.

Supplementary Table S3. Primers for real-time quantitative PCR.

Supplementary Table S4. The sequences of non-functional *StMYB113-3*, *StbHLH1-1*, and *StbHLH1-4.*

Supplementary Fig. S1. Different variants of *StAN1* presented in skin, flesh, and red vascular ring of four genotypes by qPCR melting curve analysis.

Supplementary Fig. S2. Phylogenetic relationship between Arabidopsis MYB transcription factors and anthocyanin-related MYBs of potato and other species.

Supplementary Fig. S3. Protein sequence alignment of five alleles of *StbHLH1* and one allele of *StJAF13* in differentially pigmented potatoes.

Supplementary Fig. S4. Phylogenetic relationship of anthocyanin-related *bHLH* genes of potato and other species.

Supplementary Fig. S5. Transient expression assays to probe the function of *StAN1-R0M*.

Supplementary Fig. S6. Transient expression assays to probe the function of *StAN1-R0T.*

Supplementary Fig. S7. Quantitative analysis of transcript levels of anthocyanin biosynthetic genes in transgenic tobacco leaves.

Supplementary Fig. S8. Patches of anthocyanin production in *NtJAF13 RNAi* tobacco leaves.

Supplementary Data
