# Supplementary material for: Functional diversification of the potato R2R3 MYB anthocyanin activators AN1, MYBA1, and MYB113 and their interaction with basic helix-loop-helix cofactors
Source: J Exp Bot. 2016 Feb 16;67(8):2159–76. doi: 10.1093/jxb/erw014 (PMC4809278; doi:10.1093/jxb/erw014)
Supplement: Supplementary Data [file supp_67_8_2159__index.html]

Functional diversification of the potato R2R3 MYB anthocyanin activators AN1, MYBA1, and MYB113 and their interaction with basic helix-loop-helix cofactors — Functional diversification of the potato R2R3 MYB anthocyanin activators AN1, MYBA1, and MYB113 and their interaction with basic helix-loop-helix cofactors — Supplementary Data 

# Functional diversification of the potato R2R3 MYB anthocyanin activators AN1, MYBA1, and MYB113 and their interaction with basic helix-loop-helix cofactors

## Supplementary Data

Data files

- Supplementary\_Table\_S1.pdf - Supplementary Data
- Supplementary\_Table\_S2.xlsx - Supplementary Data
- Supplementary\_Table\_S3.xlsx - Supplementary Data
- Supplementary\_Table\_S4.pdf - Supplementary Data
- Supplementary\_Figures\_S1\_S8.pdf - Supplementary Data
- Supplementary\_methods.pdf - Supplementary Data
