# Supplementary material for: Functional diversification of the potato R2R3 MYB anthocyanin activators AN1, MYBA1, and MYB113 and their interaction with basic helix-loop-helix cofactors
Source: J Exp Bot. 2016 Feb 16;67(8):2159–76. doi: 10.1093/jxb/erw014 (PMC4809278; doi:10.1093/jxb/erw014)
Supplement: Supplementary Data [file supp_erw014_Supplementary_Table_S1.pdf]

**Table S1 Summary of missense mutations found with respect to *StbHLH1* (JX848660) sequence**

| Variants of bHLH1 | Nucleotide positions | Missense mutations | Amino acid substitutions |
|---|---|---|---|
| StbHLH1-1 | 1589 | G>deletion | G→V |
| | 1597-1599 | ATA>TAG | Stop codon |
| StbHLH1-2 | | (same as StbHLH1 (JX848660)) | |
| StbHLH1-3 | 309 | G>A | T→T |
| | 646 | G>A | A→T |
| | 717 | T>C | D→D |
| | 762-767 | TGAGGA>deletion | ED→ deletion |
| | 853-867 | AATCCTATAGCGGCT>deletion | AANPI→deletion |
| | 887 | C>A | P→Q |
| | 1065 | T>A | P→P |
| | 1110 | A>T | T→T |
| StbHLH1-4 | 478-527 | GTAATAAAACTTTACCTGCAGACCCTTTGA CTTATTAATTTCCAATTATT>insertion | Stop codon |
| StbHLH1-5 | 201 | G>T | S→S |
| | 291 | G>A | L→L |
| | 315 | C>T | S→S |
| | 587 | A>C | Q→P |
| | 717 | T>C | D→D |
| | 730 | G>A | G→R |
| | 753-776 | GGATGAGGATGAGGACGAGGATGA>deletion | EDEDEDED→ deletion |
| | 817 | A>G | N→D |
| | 887 | C>A | P→Q |
| | 1174 | A>C | M→L |
| | 1264 | C>G | H→D |
| | 1360 | C>T | L→L |
| | 1365 | T>A | P→P |
| | 1393 | T>G | S→A |
| | 1404 | C>T | D→D |
| | 1419 | G>A | A→A |
| | 1688 | A>G | N→S |
| | 1696 | A>G | T→A |
| | 1710 | A>G | A→A |
| | 1714 | C>T | P→S |
| | 1733 | A>G | K→R |
| | 1743-1744 | TA>CG | ST→SA |
| | 1765 | A>G | R→G |
| | 1773 | G>A | P→P |
| | 1782 | A>C | I→I |
| | 1785 | G>C | T→T |
| | 1787 | T>C | V→A |
| | 1806 | A>G | E→E |
| | 1827 | C>G | V→V |
| | 1849 | T>C | L→L |
| | 1887 | G>A | L→L |
| | 1917 | T>A | L→L |
| | 1960 | T>C | F→L |
| | 1968 | T>G | F→L |
