# Supplementary material for: Functional diversification of the potato R2R3 MYB anthocyanin activators AN1, MYBA1, and MYB113 and their interaction with basic helix-loop-helix cofactors
Source: J Exp Bot. 2016 Feb 16;67(8):2159–76. doi: 10.1093/jxb/erw014 (PMC4809278; doi:10.1093/jxb/erw014)
Supplement: Supplementary Data [file supp_erw014_Supplementary_Table_S4.pdf]

Table S4 The sequences of non-functional *StMYB113-3*, *StbHLH1-1* and *StbHLH1-4*

> *StMYB113-3*

ATGAACTTGCCAAGTCATTGGGAGTGAGAAAAGGTTCATGGACTAAAGAAGAAGATATTCTTTTGAGGAAAT
GTATTGACAAGTATGGAGAAGGAAAGTGGCATCTTGTTCCTTCTAGAGCTGATGGTCACTTATTGCTGGGAGA
CTTCCAGGAAGAACAGCAAACGATGTGAAAAATTATTGGAACACACACCTACACAGGAAGTTAATAACCCCTC
CTCATGAGATACAAGAGAATAAGTACAATAATACCCTCAAGATCACTGAAAGCACTATACTACGACCACGACC
TCGACCTCGAACCTTCTCAAGTGCAAATAATATTTCTTGGTGCACTAACAATAGTATGATCACAAACACATTAG
ACAAAGATGACGAACAACGCAACAAAGAAATCGTAGTAAATATTTGTGAGAAGCCAACAAGAGAAACAACGT
CATCGTCTATAGACGATGACGGAGATGGAGTTAAATGGTGGACAGACTTATTGGAAAATTGGAAAGAATTTG
AGGAAGAAGCAGCAGCAGTATTGAGTTTTGAGGAAGAAAATAAGTTGTTACCAAATTTGTTGTATGAGGAAC
ATAATTCAACAACCATGCAACATGGAGAAAATGATGGTTGGGATGACTTTTCAGTTGATATTGACCTATGGAA
TCTATTTAATTAG

> *StbHLH1-1*

ATGGAGATTATACAGCCTAATAGCCTGCAGTTACAAAACATGTTGCAGAATTCCGTCCAGTCGGTTAAATGGA
CTTATAGTCTTTTTTGGCAATTTTGTCCAAAACAAGGGGTGTTAGTGTGGAGAGATGGATATTATAATGGAGC
TATAAAGACAAGGAAAACTGTGCAGCCAATGGAAGTTACTGCTGAAGAAGCTTCGCTTCATAGAAGCCAACA
ACTTAGAGAACTTTATGATTCACTTTCTGCCGGCGACTCAAATCCGCCGGCGAGAAGGCCGTCCGCAGCCTTG
TCGCCGGAGGATTTGACGGAATCCGAGTGGTTTTATCTCATGTGTGTTTCTTTCTCTTTTCCTCCACCTATTGGA
TTACCAGGCAAGGCTTATTCAAAGAAACATCACATATGGATAATGGGTGCAAATGAGGTTGATAGCAAAGTCT
TCTGTAGAGCTATTCTTGCTAAGAGTGCCCGCATACAGACTGTCGTTTGTATTCCTCTCTTGGACGGTGTAGTG
GAACTGGGAACTACAGAAAGGGTTCAAGAAGACATTGGATTCATACATCATGTAAAAAGCTTCTTCAATGAGC
AACAACAAGCTCAGCCACCAAAGCCAGCTTTATCTGAGCACTCCACTTCGGATCCCGCCGCCTTTTCGGAGCCA
CATTTTTACTCCAGCAATACTCCGTCATCCGCTGGTATTTGTCCAGCGGATCAAGATGGTAGAATTGCTGGAGA
AGAAGAAAATGAGGATGAGGATGAGGATGAGGACGAGGATGAGGATGACGAAGAAGAAAATGATGAAGTT
GAATTAGACTCGAATGGTATAGCAATTCAAAGTGTTGCTGGGGCGGCTAATCCTATAGCGGCTGAGGCTAGT
GAGCTCATGCCGCTTGATATGTCTGAAACTATACGGCTCGGCTCGCCGGATGATGGCTCTAATAATATGGATA
CTGATTTATATTTGGATGGTATTAGCCAAGCTGGAAATACGGCTGACTCTTTCAAAGCTGAGACTGCAATTAGT
TGGGCTAACTTCCAAGACCTTCAACATTTAACAGGTGTCCCTAGTTATGATGAATTATCACAAGAAGATGCACA
TTATTCTCAAACAGTTTCAGCTGTACTTGAACACCTCTCAAACACAAGTTCCAAATTTGCCTCTTCTACTACCATA
ATGGGCTCTATTTCTCCTGATTCAGCCCAATCCGCTTTCACATTATGGCCCATCATTTGTAGCCCAAATCCCTCC
CACTGCCATCGCCAACACGGCGGCGACGGCAGTGGGACCAGCTCCCAGTGGCTGCTCAAAAGCATACTATTC
ACTGTCCCATTTCTCCACACTACTAAAAAATTACACCAACTACCTGAAGCTCTTTCTCCAAAGTCACGAGACTCT
GCGGCCGACTCTTCAGCCGCAGCGTCTCGATTCCGCAAAGGATGTACGATAAATAGTAGTACACAACAAGAA
GAGACGAATGGGAACCATGTACTAGCGGAACGACGTCGTAGAGAGAAGCTGAATGAGCGTTTTATTATTTTA
AGGTCCTTAGTACCGTTCGTTACGAAAATGGATAAAGCATCGATACTTGTGATACAATAGAGTATGTGAAGCA
GTTACGTAAGAAAGTTCAGGATCTTGAAGCGCGTGATCGTCACGCGGAAACCACAAAAAATGCAGATGAAAA
GAGTGGTACAGCAACAGTGAAGGCGTTTTCGGGGAAGGGTAAGAGGAGAATGAAGAGTGCGGTGGAGGGA
AGTATAGGCGGAGCACCAGCAAAGATCACCGCGTCTCCACCTATGGATGAGGAAGTTTTGCAAGTGGAAGTC
TCAATCATCGAAAACGATGCACTGGTGGAGCTCCGGTGTCCGTACAAAGAAGGGTTGTTGTTAGATGTAATGC
AAATGCTAAGGGAACTAAAAGTGGAAGTTGTAGCAATCCAATCATCTCTTAATACTGGCCTCTTATTGGCTGA
GTTAAGAGCTAAGGTAAAAGAAGATATATATGGAAGGAAAGCAAGCATTTTGGAAGTGAAAAAGTCAATACA
TCAGATAATCCCTAGAGCTAATTAA

> *StbHLH1-4*

ATGGAGATTATACAGCCTAATAGCCTGCAGTTACAAAACATGTTGCAGAATTCCGTCCAGTCGGTTAAATGGA
CTTATAGTCTTTTTTGGCAATTTTGTCCAAAACAAGGGGTGTTAATGTGGAGAGATGGATATTATAATGGAGCT
ATAAAGACAAGGAAAACTGTGCAGCCAATGGAAGTTACTGCTGAAGAAGCTTCTCTTCATAGAAGCCAACAA
CTTAGAGAACTTTATGATTCACTTTCTGCCGGCGACTCAAATCCGCCGGCGAGAAGGCCGTCCGCAGCCTTATC
GCCGGAGGATTTGACGGAATCTGAGTGGTTTTATCTCATGTGTGTTTCTTTCTCTTTTCCTCCACCTATTGGATT

ACCAGGCAAGGCTTATTCAAAGAAACATCACATATGGATAATGGGTGCAAATGAGGTTGATAGCAAAGTCTT
CTGTAGAGCTATTCTTGCTAAGAGTGCCCGCATACAGGTAATAAAACTTTACCTGCAGACCCTTTGACTTATTA
ATTTCCAATTATTACTGTCGTTTGTATTCCTCTCTTGGACGGTGTAGTGGAACTGGGAACTACAGAAAGGGTTC
AAGAAGACATTGGATTCATACATCATGTAAAAAGCTTCTTCAATGAGCCACAACAAGCTCAGCCACCAAAGCC
AGCTTTATCTGAGCACTCCACTTCGGATCCCGCCGCCTTTTCGGAGCCACATTTTTACTCCAGCAATACTCCGTC
ATCCGCTGGTATTTGTCCAGCGGATCAAGACGGTAGAATTGCTAGAGAAGAAGAAAATGAGGATGAGGATG
ACGAAGAAGAAAATGATGAAGTTGAATTAGACTCGGATGGTATAGCAATTCAAAGTGTTGCTGGGGCGGCTA
ATCCTATAGCGGCTGAGGCTAGTGAGCTCATGCAGCTTGATATGTCTGAAACTATACGGCTCGGCTCGCCGGA
TGATGGCTCTAATAATATGGATACTGATTTATATTTGGATGGTATTAGCCAAGCTGGAAATACGGCTGACTCTT
TCAAAGCTGAGACTGCAATTAGTTGGGCTAACTTCCAAGACCTTCAACATTTAACAGGTGTCCCTAGTTATGAT
GAATTATCACAAGAAGATGCACATTATTCTCAAACAGTTTCAGCTGTACTTGAACACCTCTCAAACACAAGTTC
CAAATTTGCCTCTTCTACTACCATACTGGGCTCTATTTCTCCTGATTCAGCCCAATCCGCTTTCACATTATGGCCC
ATCATTTGTAGCCCAAATCCCTCCCACTGCCATCGCCAACACGGCGGCGACGGCAGTGGGACCAGCTCCCAGT
GGCTGCTCAAAAGCATACTATTCACTGTCCCATTTCTCCACACTACTAAAAAATTACACCAACTACCTGAAGCTC
TTTCTCCAAAGTCACGAGACTCTGCGGCCGACTCTTCAGCCGCAGCGTCTCGATTCCGCAAAGGATGTACGAT
AAATAGTAGTACACAACAAGAAGAGACGAATGGGAACCATGTACTAGCGGAACGACGTCGTAGAGAGAAGC
TGAATGAGCGTTTTATTATTTTAAGGTCCTTAGTACCGTTCGTTACGAAAATGGATAAAGCATCGATACTTGGT
GATACAATAGAGTATGTGAAGCAGTTACGTAAGAAAGTTCAGGATCTTGAAGCGCGTGATCGTCACGCGGAA
ACCACAAAAAATGCAGATGAAAAGAATGGTACAACAATAGTGAAGGCATTTCCGGGGAAGGGTAAGAGGAA
AATGAAGAGTACGGTGGAGGGAAGTATAGGCAGAGCACCGGCAAAGATAACGGTGTCTCCACCTATGGATG
AAGAAGTTTTGCAAGTGGAAGTCTCAATCATCGAAAACGATGCATTGGTGGAGCTCCGGTGTCCGTACAAAG
AAGGGTTGTTGTTAGATGTAATGCAAATGCTAAGGGAACTTAAAGTGGAAGTTGTAGCAATCCAATCATCTCT
TAATACTGGCTTCTTATTTGCTGAGTTAAGAGCTAAGGTAAAAGAAGATATATATGGAAGGAAAGCAAGCATT
TTGGAAGTGAAAAAGTCAATACATCAGATAATCCCTAGAGCTAATTAA
