# Supplementary material for: Functional diversification of the potato R2R3 MYB anthocyanin activators AN1, MYBA1, and MYB113 and their interaction with basic helix-loop-helix cofactors
Source: J Exp Bot. 2016 Feb 16;67(8):2159–76. doi: 10.1093/jxb/erw014 (PMC4809278; doi:10.1093/jxb/erw014)
Supplement: Supplementary Data [file supp_erw014_Supplementary_Figures_S1_S8.pdf]

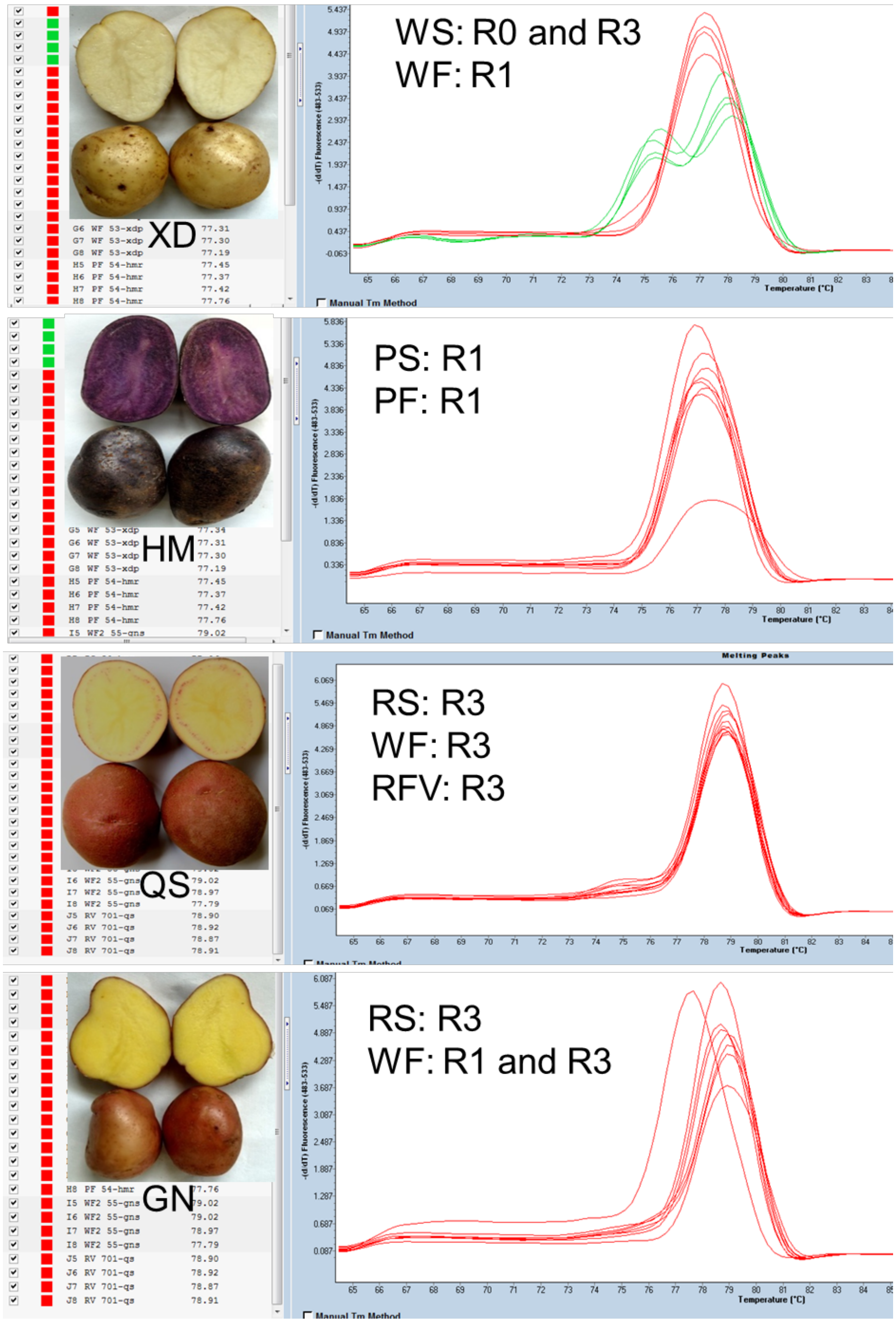

Supplementary Fig. S1. Different variants of *StAN1* presented in skin, flesh and red vascular ring of four genotypes by qPCR melting curve analysis. A pair of primers (StAN1-5F/StAN1-6R) flanking R repeat region was used to identify variants. Green lines indicate two PCR products were detected, red lines indicate single PCR product.

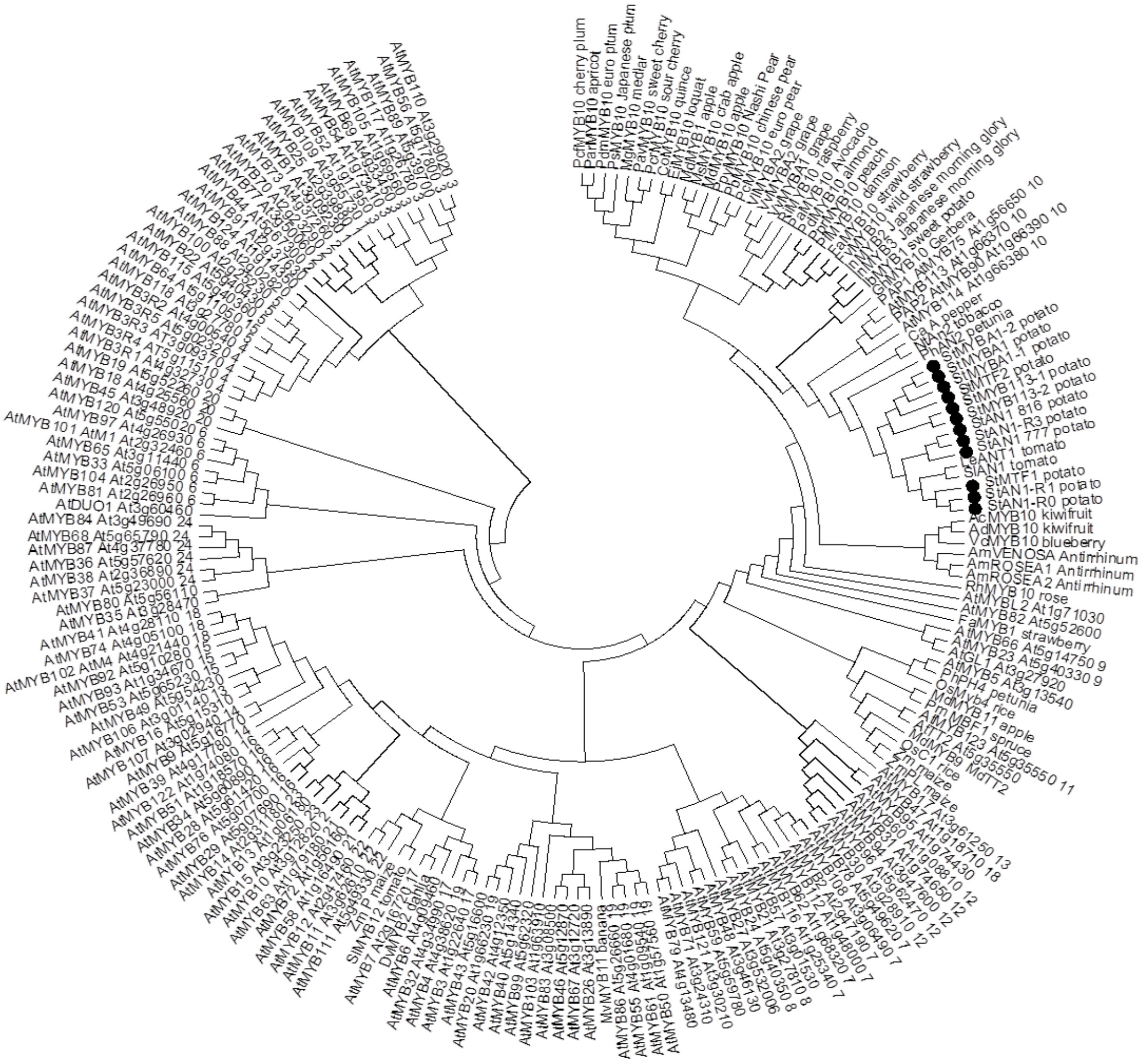

Supplementary Fig. S2. Phylogenetic relationship between Arabidopsis MYB transcription factors and anthocyanin-related MYBs of potato and other species.

Potato *StAN1-R0*, *StAN1-R1*, *StAN1-R3*, *StMYBA1-1*, *StMYBA1-2*, *StMYB113-1*, *StMYB113-2* cluster within the anthocyanin MYB regulator subgroup.

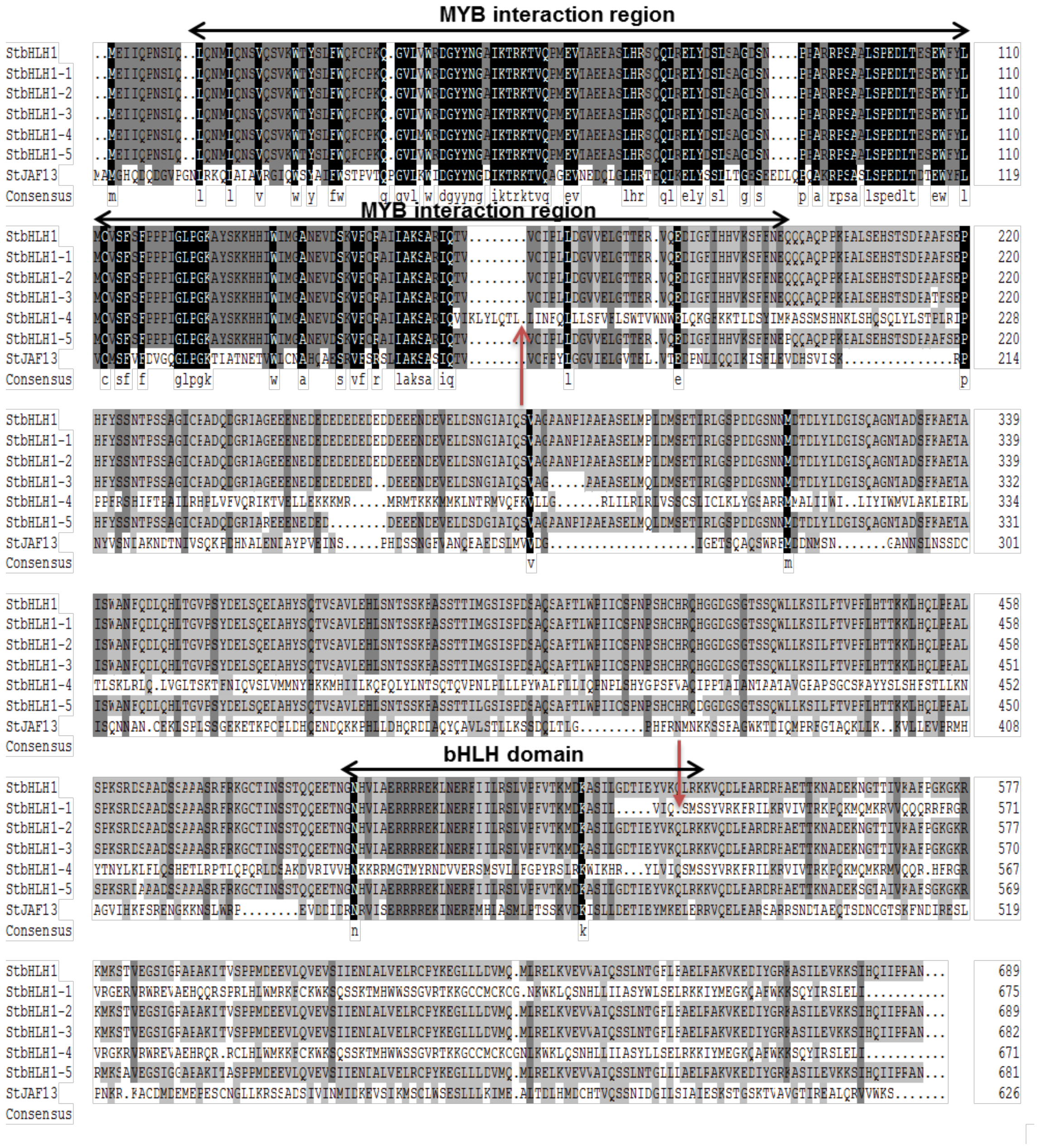

Supplementary Fig. S3. Protein sequence alignment of five alleles of StbHLH1 and one allele of StJAF13 in differentially pigmented potatoes. The MYB interaction region and bHLH domain are indicated by arrows above. NCBI Protein accession numbers: StbHLH1: AGC31677; StbHLH1-2 KP317173; StbHLH1-3 KP317174; StbHLH1-5 KP317175; StJAF13 KP317176. The premature stop codons of StbHLH1-1 and StbHLH1-4 were indicated by red arrows.

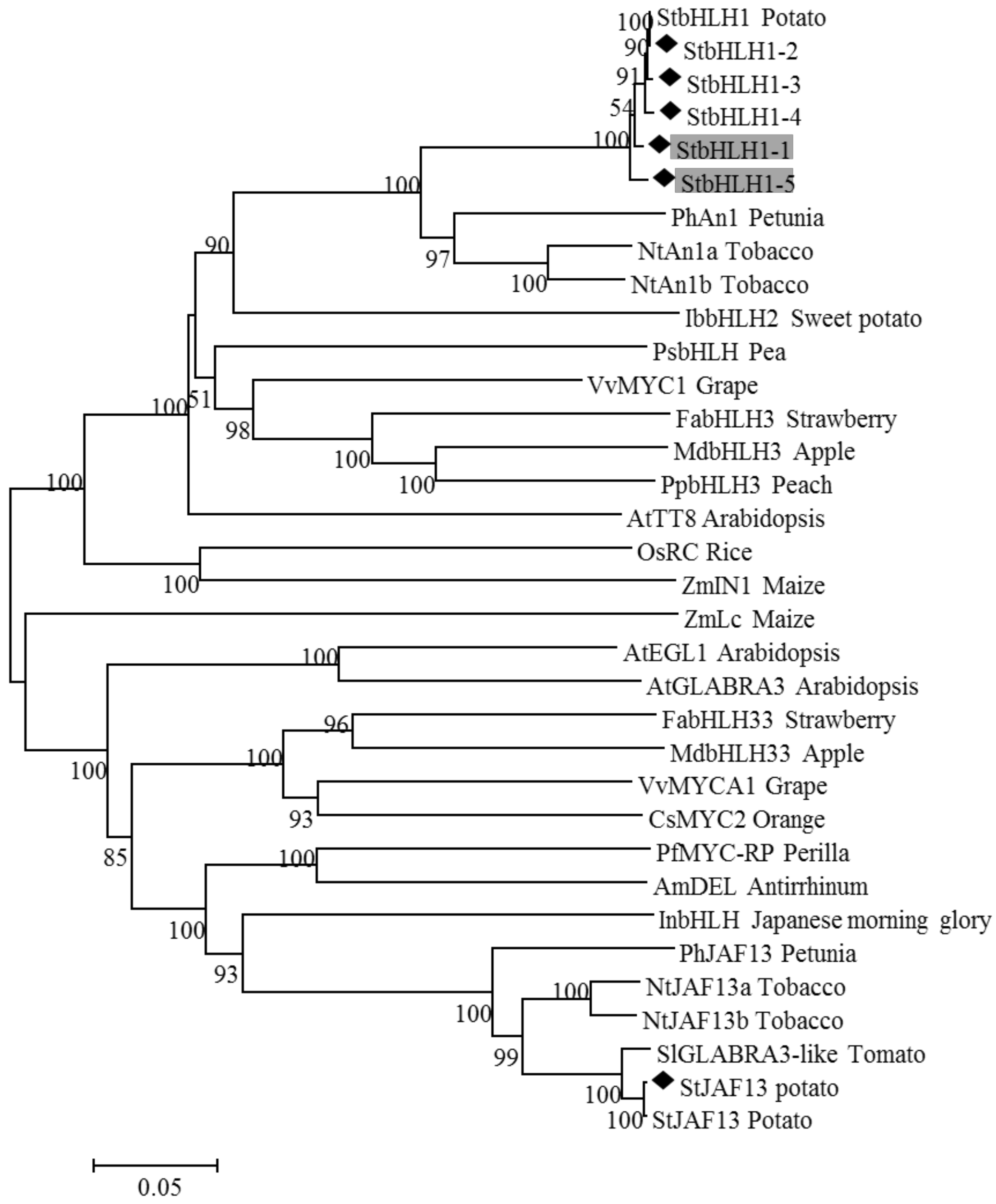

Supplementary Fig. S4. Phylogenetic relationship of anthocyanin-related *bHLH* genes of potato and other species. Sequences were aligned using Geneious v.6.1.6 (Drummond *et al.*, 2011) with a cost matrix of 65%, a gap open penalty of 12 and a gap extension penalty of 3. Phylogenetic and molecular evolutionary analysis was conducted using MEGA version 6.0 [the evolutionary history was inferred using the neighbour-joining method and 1000 bootstrap replicates, bootstrap values less than 50 are not shown], *StbHLH1-1*, *StbHLH1-2*, *StbHLH1-3*, *StbHLH1-4*, *StbHLH1-5* and *StJAF13* are indicated by black diamond. The non-functional *StbHLH1-1* and *StbHLH1-4* are indicated by grey box.

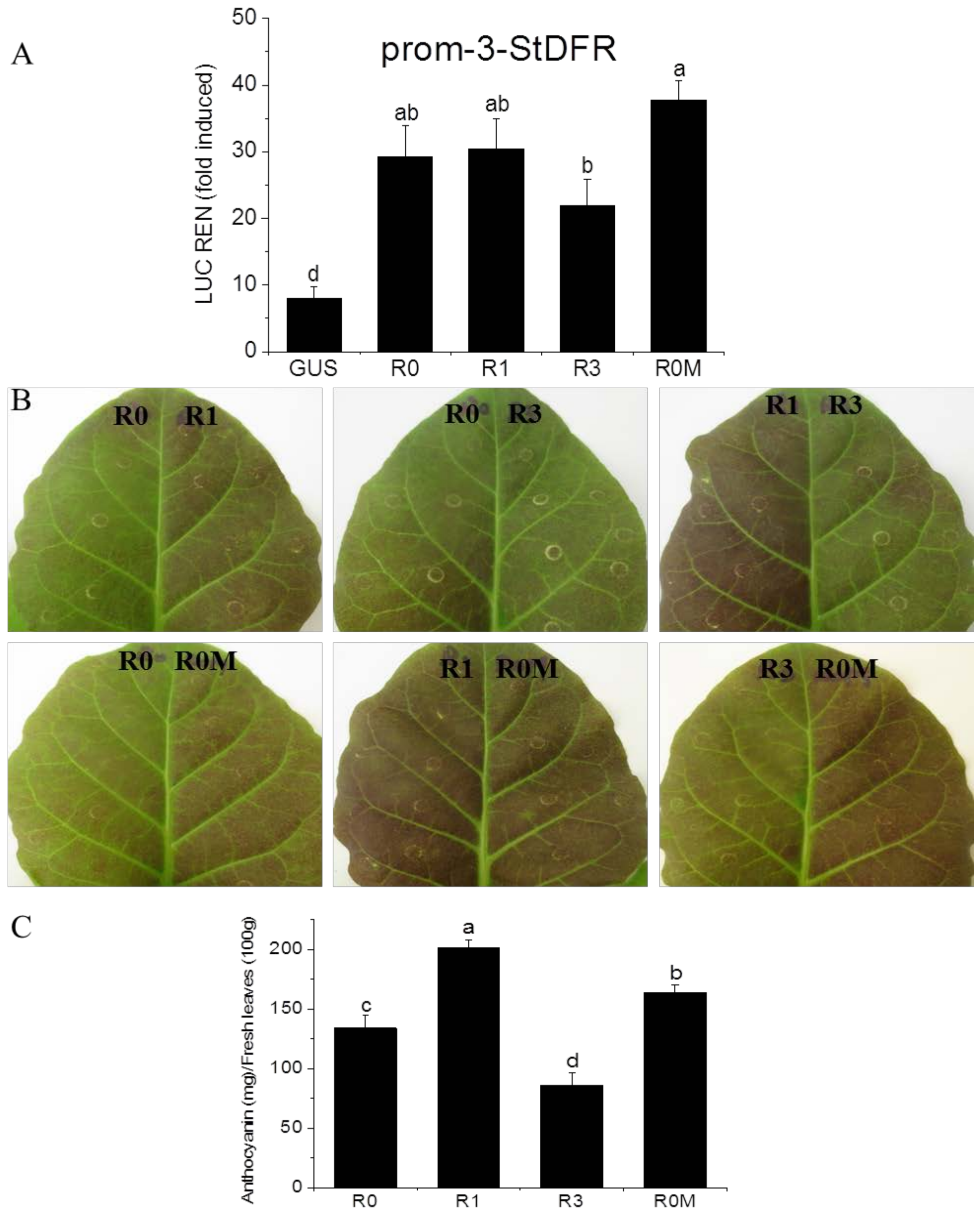

Supplementary Fig. S5. Transient expression assays to probe the function of *StAN1-R0M*. (A) The activity of the *prom-3-StDFR* promoter affected by the *StAN1-R0*, *StAN1-R1*, *StAN1-R3* and *StAN1-R0M*. Error bars are the SE of three independent experiments with four replicate reactions. (B) Patches of anthocyanin production in tobacco leaves by *StAN1-R0*, *StAN1-R1*, *StAN1-R3* and *StAN1-R0M.* Pictures were taken at 4-d after infiltration. (C) Anthocyanin was extracted from patches described in (B), Error bars are the SE of three biological replicates. Statistical significance was determined by one-way ANOVA; significant differences between means (LSD, $P < 0.05$) are indicated where letters (a, b, c, etc.) above the bar differ.

A

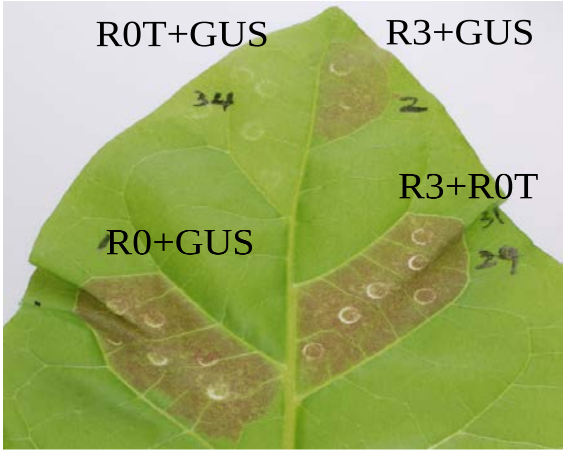

B

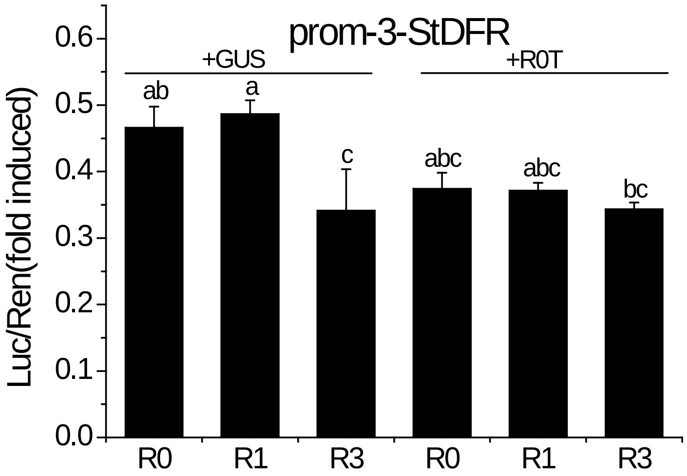

Supplementary Fig. S6. Transient expression assays to probe the function of *StAN1-R0T*. (A) Patches of anthocyanin production in *NtAN1 RNAi* tobacco leaves by the combinations as shown. Pictures were taken at 4-d after infiltration. (B) The activity of the *prom-3-StDFR* promoter affected by the *StAN1-R0*, *StAN1-R1*, *StAN1-R3* either combined with GUS or R0T. Error bars are the SE of three independent experiments with four replicate reactions. Statistical significance was determined by one-way ANOVA; significant differences between means (LSD, $P < 0.05$) are indicated where letters (a, b, c, etc.) above the bar differ.

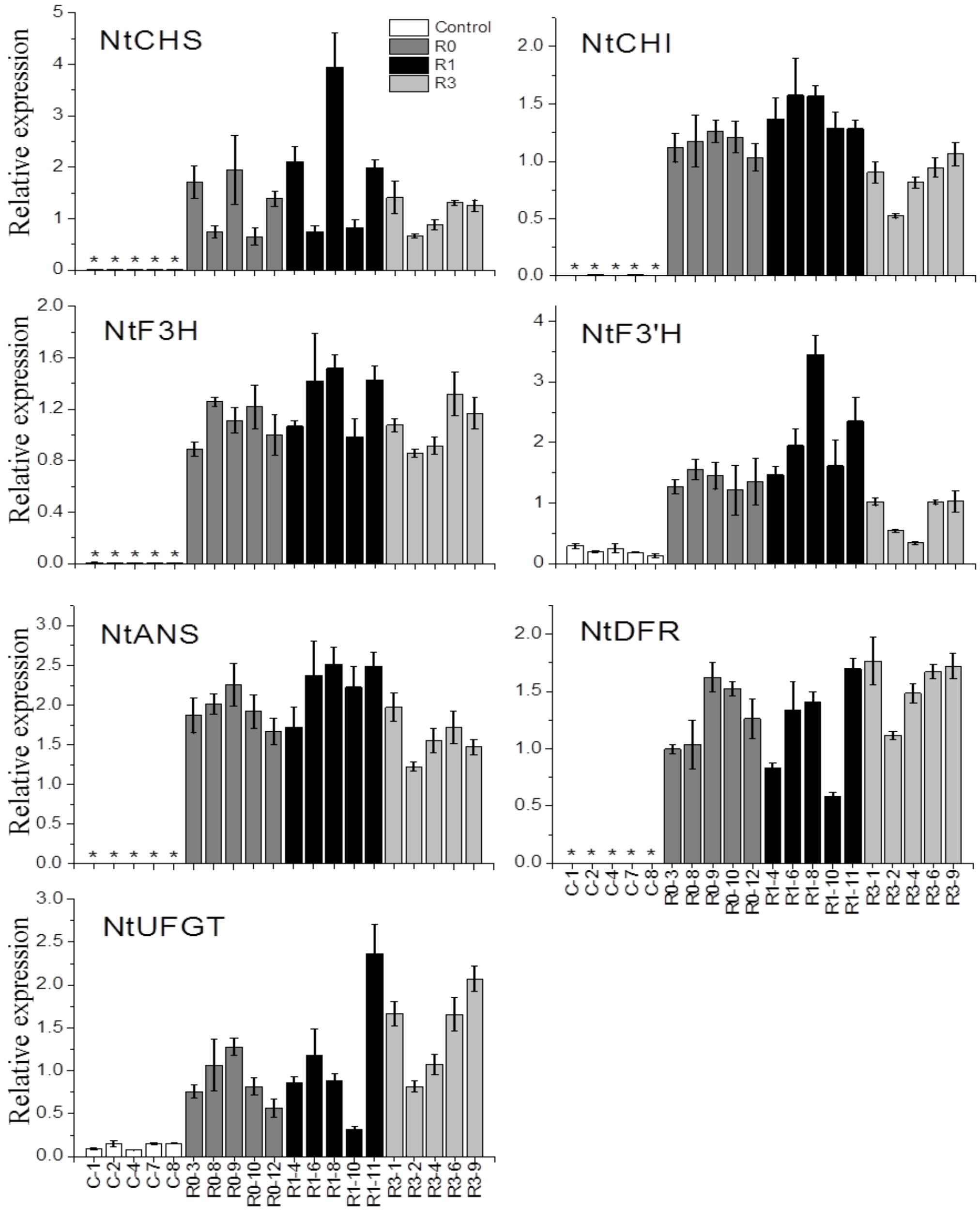

Supplementary Fig. S7. Quantitative analysis of transcript levels of anthocyanin biosynthetic genes in transgenic tobacco leaves carrying 35S:empty vector pSAK277 (Control) and overexpressing *StAN1-R0*, *StAN1-R1* and *StAN1-R3*. Error bars are the SE for four replicates per line. Statistical significance was determined by one-way ANOVA; significant differences between means (LSD, $P < 0.05$) are indicated where letters (a, b, c, etc.) above the bar differ. Genotypes denoted by * showed no detectable levels of expression.

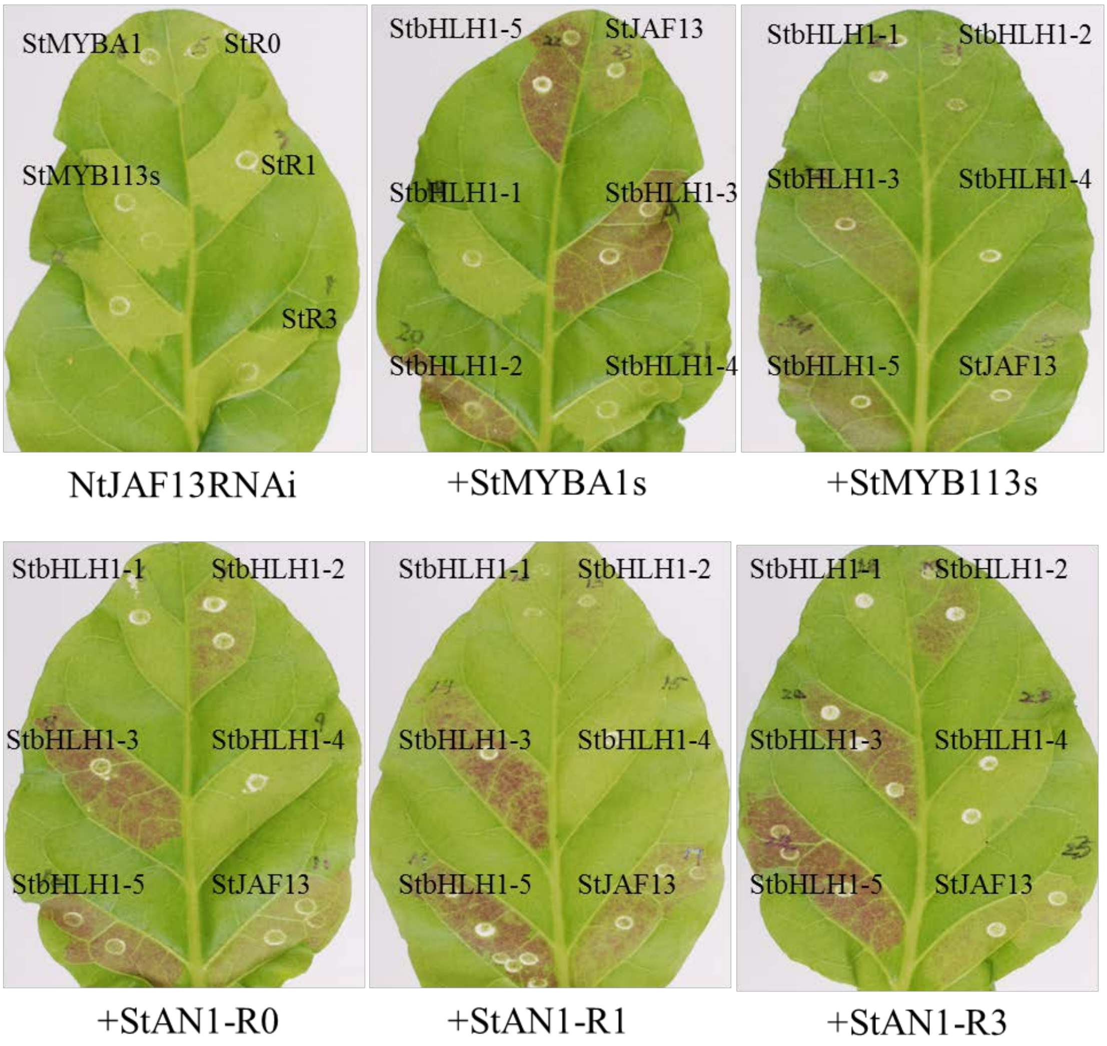

Supplementary Fig. S8. Patches of anthocyanin production in *NtJAF13 RNAi* tobacco leaves by *StMYBA1s*, *StMYB113s*, *StAN1-R0* , *StAN1-R1*, *StAN1-R3* alone or combined with five variants of *StbHLH1* and *StJAF13*, respectively.
