# Supplementary material for: Functional diversification of the potato R2R3 MYB anthocyanin activators AN1, MYBA1, and MYB113 and their interaction with basic helix-loop-helix cofactors
Source: J Exp Bot. 2016 Feb 16;67(8):2159–76. doi: 10.1093/jxb/erw014 (PMC4809278; doi:10.1093/jxb/erw014)
Supplement: Supplementary Data [file supp_erw014_Supplementary_methods.pdf]

Supplementary methods

Gene cloning and sequence analysis

Full-length coding sequences of three alleles of the potato R2R3 MYB *StAN1*, termed as *StAN1-R0*, *StAN1-R1* and *StAN1-R3*, two alleles of the R2R3 MYB *StMYBA1*, termed as *StMYBA-1* and *StMYBA-2*, and three alleles of the R2R3 MYB *StMYB113*, termed as *StMYB113-1*, *StMYB113-2* and *StMYB113-3*, five alleles of *StbHLH1*, termed as *StbHLH1-1*, *StbHLH1-2*, *StbHLH1-3*, *StbHLH1-4* and *StbHLH1-5*, and one allele of *StJAF13* were amplified from cDNA of skin and flesh of four cultivars using Platinum *Taq* DNA Polymerase High Fidelity (Invitrogen, USA) based on the potato *StAN1* sequence (AY841129) with primers StAN1-F and StAN1-R, *StMYBA1* sequence (JQ219855) with primers StMYBA1-F and StMYBA1-R, StMYB113 sequence (OkPScomp56435_c3_seq8, KU242748) with primers StMYB113-F and StMYB113-R, *StbHLH1* sequence (JX848660) with primers StbHLH1-F and StbHLH1-R and *StJAF13* (HG763862) with primers StJAF13-F and StJAF13-R. Full-length fragments of *StAN1-R0*, *StAN1-R1*, *StAN1-R3*, *StMYBA-1*, *StMYBA-2*, *StMYB113-1*, *StMYB113-2* and *StMYB113-3* were released from pGEM-T Easy and ligated into the binary vectro pSAK277 with a 35S promoter by using *Eco*RI restriction site.

Full-length fragments of *StbHLH1s* and *StJAF13* were ligated into the binary vector pHEX2 with a 35S promoter by using the pENTR Directional TOPO cloning kit. The promoters of potato *DFR* (*Prom-1-StDFR*, 1.792 kb upstream of *DFR*, Genbank: KM873036; *Prom-2-StDFR*, 1.733 kb upstream of *DFR*, Genbank: KM873037; *Prom-3-StDFR*, 1.99 kb upstream of *DFR*, Genbank: KM873038) and F3’5’H (2.03kb upstream of *F3’5’H*, Genbank: KM873038) were isolated from the four potato cultivars and inserted into the cloning site of pGreenII 0800-LUC vector and modified to introduce a *Nco*I site at the 3’ end of the sequence, allowing the promoter to be cloned as a transcriptional fusion with the firefly luciferase gene (LUC). LUC activity

relative to REN was expressed as a ratio to show activation of the promoter by a transcription factor included in another plasmid.

Clones were confirmed by sequencing (Macrogen, Korea), and the sequences were aligned using Vector NTI (Invitrogen, USA). Sequences for *StAN1-R0, StAN1-R1*, *StAN1-R3*, *StMYBA1-1*, *StMYBA1-2*, *StMYB113-1*, *StMYB113-2*, *StbHLH1-2*, *StbHLH1-3*, *StbHLH1-5* and *StJAF13* were submitted to GenBank (the GenBank accession number are KM822778, KM822779, KM822780, KP317177, KP317178, KP317179, KP317180, KP317173, KP317174, KP317175 and KP317176 respectively).

Transient assays of gene function

The promoters of potato *DFR* and *F3'5'H*, transcription factors *StAN1-R0, StAN1-R1*, *StAN1-R3*, *StMYBA1-1*, *StMYBA1-2*, *StMYB113-1*, *StMYB113-2* and *StMYB113-3* in pSAK277, *StbHLH1-1*, *StbHLH1-2*, *StbHLH1-3*, *StbHLH1-4*, *StbHLH1-5* in pHEX2, *StJAF13* in pHEX2 and the negative control GUS in pSAK277 were used in transient assays.

Nicotiana benthamiana plants were grown under glasshouse conditions until five to seven leaves were available for infiltration with *Agrobacterium*. Approximately 300μl of *Agrobacterium* culture containing genes of interest was infiltrated at four points into a young leaf. At three days post infiltration, 3 mm leaf discs were cut with a hole-puncher and placed into wells of a 96-well-plate containing 50 μl of PBS (phosphate buffered saline) with 4 replicates from each plant. LUC and REN activities were measured and analyzed by using an VICTOR3 Multilabel Readers (Perkin Elmer, Boston, MA, USA).
